# Compositional engineering of phase-stable and highly efficient deep-red emitting phosphor for advanced plant lighting systems

**DOI:** 10.1038/s41377-024-01679-9

**Published:** 2024-12-11

**Authors:** Jianwei Qiao, Dehong Li, Qiufeng Shi, Haijie Guo, Ping Huang, Lei Wang

**Affiliations:** College of Physics and Optoelectronic Engineering, Taiyuan University of Technology, Taiyuan, 030024 China

**Keywords:** Optical properties of diamond, Inorganic LEDs

## Abstract

Inorganic luminescent materials hold great promise for optoelectronic device applications, yet the limited efficiency and poor thermal stability of oxide-based deep-red emitting phosphors hinder the advancement of plant lighting technologies. Herein, a simple compositional engineering strategy is proposed to stabilize the phase, boost external quantum efficiency (EQE) and enhance thermal stability. The chemical modification of the PO_4_ tetrahedron in NaMgPO_4_:Eu by incorporating SiO_4_ lowers the formation energy, leading to the generation of pure olivine phase and increasing the EQE from 27% to 52%, setting a record for oxide deep-red phosphors. In parallel, the introduced deep defect level improves thermal stability at 150 °C from 62.5% to 85.4%. Besides, the excitation and emission peaks shifted to 440 nm and 675 nm, respectively, aligning precisely with the specific spectral absorption requirements of plant phytochromes. Moreover, the luminescent intensity showed nearly no decay after being exposed to 80% relative humidity and 80 ^o^C for 6 h, and the pc-LED utilizing Na_1.06_MgP_0.94_Si_0.06_O_4_:Eu achieves a high output power of 780 mW at 300 mA. Our research demonstrates a facile method for optimizing the performance of inorganic luminescent materials and provides alternative solutions for low-cost plant lighting.

## Abstract (web summary)

We synthesized pure olivine phase Na_1.06_MgP_0.94_Si_0.06_O_4_:Eu deep-red phosphor using straightforward compositional engineering. This phosphor attains a remarkable EQE value of 52%, surpassing the previous record for oxide-based deep-red phosphors, while maintaining a high thermal stability of 85.4% at 150 °C.

## Graphical abstract

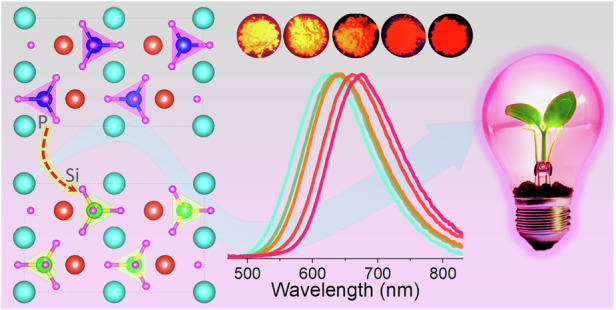

## Introduction

The plant factory has gained considerable attention as a solution for food production amidst the worsening effects of climate change, population challenges, and rising costs of traditional agricultural methods^1,2^. Light is crucial for the life activities of plants, as it regulates plant metabolism by controlling plant germination, flowering, and accelerating stem and leaf growth^3–5^. The process of photosynthesis in plants primarily depends on the absorption of plant pigments (such as chlorophyll a, chlorophyll b, carotenoids, phytochrome P_R_, and phytochrome P_FR_) within the blue (440 nm), deep-red (660 nm), and far-red (730 nm) regions^6,7^. At present, the commercial plant lighting devices constructed using blue and red diode chips suffer from high expenses and limited electroluminescent bands that fail to fully cover the absorption area of phytochromes^8^. As a promising alternative, phosphor-converted light-emitting diodes (pc-LEDs) appear to be the most straightforward, cost-effective, and convenient plant lighting strategy. Thus, developing efficient phosphors that can be well matched with photoreceptors becomes key to achieving low-cost indoor agricultural cultivation.

Numerous endeavors have been directed towards discovering deep-red phosphors by doping transition metal ions (Cr^3+^, Mn^4+^) or rare earth ions (Pr^3+^, Yb^3+^) into inorganic host materials^9,10^. However, the sharp emission lines of Mn^4+^, Pr^3+^ and Yb^3+^ ions can hardly cover both the P_R_ and P_FR_ regions simultaneously, and excitation spectra of these phosphors struggle to match with blue chips^11–14^. Recently, Cr^3+^-activated phosphors have garnered significant interest because of their broad far-red or near-infrared emission^15,16^. Nevertheless, the spin-forbidden *d*-*d* transitions of Cr^3+^ activators lead to weak absorption and low quantum yield (EQE < 40%)^17–19^. In contrast, Eu^2+^ activated phosphors are typically endowed with strong absorption and high EQE, making them extensively utilized in solid-state lighting applications^20–22^. For instance, the broad deep-red emission band of the widely recognized nitride phosphor CaAlSiN_3_:Eu^2+^ effectively overlaps with absorption spectra of plant pigments, rendering it an ideal luminescent material for plant lighting^8,23^. Nonetheless, the water/oxygen-free preparation environment of precursors, along with high pressure (~1 MPa) and high temperature (>2000 K) conditions necessary for sintering process, result in high production costs for nitride phosphors^24^. Oxide-based phosphors like the commercial YAG:Ce, BaMaAl_10_O_17_:Eu^2+^ and Ba_2_SiO_4_:Eu^2+^ have emerged as excellent choices for affordable pc-LEDs. Unfortunately, only a limited number of Eu^2+^-activated oxide deep-red phosphors have been discovered to date. Examples like Ca_1.2_Eu_0.8_SiO_4_ and Ca_3.93_Eu_0.07_(PO_4_)_2_O, demonstrate low emission efficiency (EQE < 40%) and poor thermal stability (<80%@150 °C)^25^. It is noteworthy that Hasegawa and Ishigaki et al. developed red-emitting phosphors of olivine phase NaMgPO_4_:Eu^2+^ (O-NaMgPO_4_) with an IQE up to 80% using a rapid quenching method^26,27^. Recently, Wang et al. synthesized Na(Mg,Zn)PO_4_:Eu red phosphor with an EQE of 42% through Zn-Mg solid solution^28^. However, these studies failed to obtain a completely pure O-NaMgPO_4_ phase, and the emission peak (620 nm) did not fall within the optimal deep-red region (660 nm), in addition to the low EQE value (<45%) resulting from the low absorption coefficient in the blue light region. Hence, there is still an urgent need to discover oxide deep-red emitting phosphors with high efficiency and exceptional thermal stability.

In this work, we successfully achieved multiple goals via straightforward composition engineering, creating a phase-stable, efficient, and thermally stable deep-red phosphor Na_1.06_MgP_0.94_Si_0.06_O_4_:Eu. As a result, the successful incorporation of Si^4+^ ions significantly reduced the formation energy, overcoming the challenge of synthesizing pure olivine phase through traditional high-temperature solid-state method. By modulating the sites occupation of Eu^2+^ ions, the absorption in the blue region is significantly enhanced, and the emission color turned from red to deep-red region. The increase in the number of luminescent centers of Eu^2+^ leads to an enhancement of EQE to 52%, while the creation of deeper defect levels improves thermal stability to 85.4%@150 °C. Moreover, the lettuce under irradiation of artificial pc-LEDs exhibits significant growth advantages compared to that of non-illuminated sample, suggesting the substantial potential application of Na_1.06_MgP_0.94_Si_0.06_O_4_:Eu in the low-cost lighting for indoor agriculture.

## Results

### Phase stabilization of O-NaMgPO_4_

**Fig. 1:**
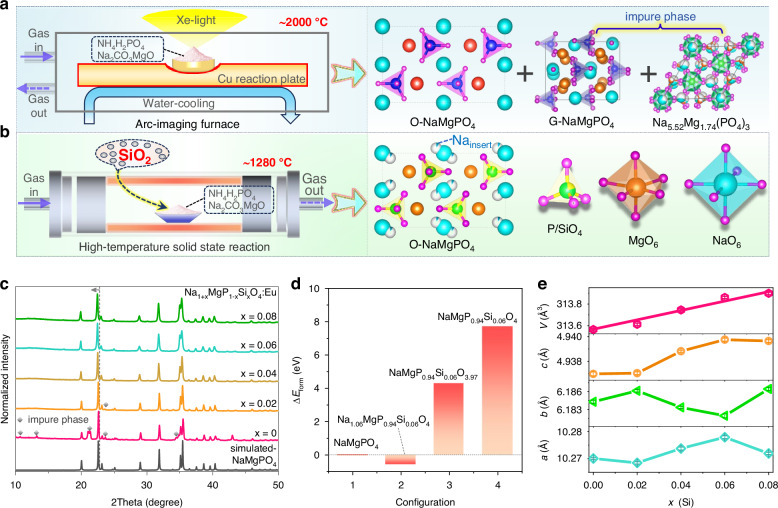
Synthesis method and structural characterization of olivine-type NaMgPO_4_:Eu^2+^ phosphor. **a** Schematic image of arc-imaging furnace and **b** traditional high-temperature solid state method, as well as the crystal structure of the synthesized products. **c** X-ray powder diffraction patterns of Na_1+*x*_MgP_1-*x*_Si_*x*_O_4_:Eu^2+^ (*x* = 0–0.08) phosphors and the simulated pattern profile based on Rietveld refinement results. **d** Differences in formation energies for four different configurations calculated by using First-Principle calculations based on DFT. **e** Dependence of the lattice parameters (*a*, *b*, *c*, *V*) on the doping concentration of Si

The olivine-type NaMgPO_4_:Eu^2+^ (O-NaMgPO_4_:Eu^2+^) was initially discovered using a so-called arc-imaging furnace method (Fig. 1a), enabling a rapid temperature increase up to 2000 °C followed by quick cooling using a water-cooling system^27^. However, the synthesized samples contain many impure phases such as glaserite-type NaMgPO_4_ (G-NaMgPO_4_) and Na_5.52_Mg_1.74_(PO_4_)_3_, as shown in Fig. 1a and Supplementary Fig. S1a^29^. Although the olivine phase in NaMgPO_4_ was later stabilized through the “melting and quenching process”, it still contains a number of unidentified impure phases (Supplementary Fig. S1b)^26^. Figure 1b depicts the schematic diagram of synthesizing pure-phase O-NaMgPO_4_:Eu^2+^ phosphor in this research through traditional high-temperature solid-state reaction (HTSSR) by incorporating silicon into the synthetic raw materials. In comparison to the previous two methods, this approach offers the benefits of simplicity, affordability, and large-scale synthesis of O-NaMgPO_4_:Eu^2+^. Figure 1c shows the simulated pattern of O-NaMgPO_4_ and the XRD patterns of Na_1+*x*_MgP_1-*x*_Si_*x*_O_4_:Eu (*x* = 0–0.08) samples synthesized using HTSSR method. Obviously, the XRD pattern of the undoped Si sample (*x* = 0) contains multiple diffraction peaks of impure phases, among which the G-NaMgPO_4_ impurity leads to the generation of blue luminescent particles in the orange powder (Supplementary Fig. S2a). With the increase of *x* value, the impure peaks gradually diminish, and when *x* ≥ 0.04, all diffraction peaks can be well matched with the simulated pattern (Fig. 1c). This indicates that the introduction of Si effectively stabilizes the olivine phase and achieves completely pure Na_1+*x*_MgP_1-*x*_Si_*x*_O_4_:Eu (*x* = 0.04–0.08) phosphors. To delve deeper into the phase stability mechanism, we subsequently investigated the variations in formation energies (Δ*E*_form_) across four different configurations that may exist after the introduction of Si, including NaMgPO_4_ (initial configuration), NaMgP_0.94_Si_0.06_O_4_ (P^5+^→Si^4+^), Na_1.06_MgP_0.94_Si_0.06_O_4_ (P^5+^→Si^4+^ with additional Na occupying interstitial positions), and NaMgP_0.94_Si_0.06_O_3.97_ (P^5+^→Si^4+^ leading to the creation of oxygen vacancy defects). As exhibited in Fig. 1d, the negative formation energy of Na_1.06_MgP_0.94_Si_0.06_O_4_ (Δ*E*_form_ = −0.58 eV) suggests that the P^5+^→Si^4+^ substitution effectively reduces the formation energy and promotes the formation of the olivine phase, accompanied by the generation of interstitial Na sites.

To analyze the changes in crystal structure after Si doping, Rietveld refinements were performed on all samples, as shown in Supplementary Fig. S2. Considering the difference in ionic radii, we take O-NaMgPO_4_ as a prototype and assume that Si^4+^ occupies the P^5+^ site and the additional Na enters an interstitial site. Trace amount of Eu^2+^ was disregarded for structure determination due to its insignificant impact on the scattering density. Final refinement of this model was stable with low R-factors (Supplementary Table S1). The parameters of atoms’ coordinates and main bond lengths of polyhedra are listed in Supplementary Tables S2 and 3 for comparison. The cell volume V exhibits a linear increase with *x* values due to the larger ionic radius of Si^4+^ (CN = 4, 0.26 Å) compared to P^5+^ (CN = 4, 0.17 Å), as depicted in Fig. 1e and Supplementary Table S4. This also clarifies why the XRD diffraction peaks in Fig. 1c shift towards smaller angles. The P/SiO_4_ tetrahedra and MgO_6_ octahedra connect to each other, creating a closely packed structure, with Na atoms locate in quadrilateral channels. As for the extra Na atoms, they occupy a second Wyckoff position corresponding to the displacement of the Na(1) sites (Fig. 1b), analogous to the structural alterations induced by P^5+^→Si^4+^ substitution in Na_3+*x*_Sc_2_Si_*x*_P_3-*x*_O_12_^30^. Scanning electron microscope (SEM) images of NaMgPO_4_ and Na_1.06_MgP_0.94_Si_0.06_O_4_:Eu (Supplementary Fig. S3) reveal a smooth surface with a particle size of about 10 μm, demonstrating its high crystallinity. The EDS elemental mapping reveals that Na, Mg, P, Si and O are homogenously dispersed in the host (Supplementary Fig. S3f), and the detected mole ratio for each element is close to the stoichiometric ratio of Na_1.06_MgP_0.94_Si_0.06_O_4_ (Supplementary Fig. S4). To further substantiate the above inference, the Raman spectra of NaMgPO_4_ and Na_1.06_MgP_0.94_Si_0.06_O_4_:Eu are presented in Supplementary Fig. S5a. Except the obvious Raman modes from PO_4_^3-^ group, Na_1.06_MgP_0.94_Si_0.06_O_4_:Eu also exhibits a small peak at 855 cm^−1^ originating from the SiO_4_^4-^ group^31,32^. Moreover, compared to NaMgPO_4_, the Si-2p XPS spectra of Na_1.08_MgP_0.92_Si_0.08_O_4_:Eu and Na_1.06_MgP_0.94_Si_0.06_O_4_:Eu show a broad asymmetric band centered at 102 eV corresponding to Si^4+^ ions (Supplementary Fig. S5b)^33^. All the above results suggest that Si successfully enter NaMgPO_4_ lattice and form P/SiO_4_ tetrahedra.

### Tuning PL and PLE spectra

**Fig. 2:**
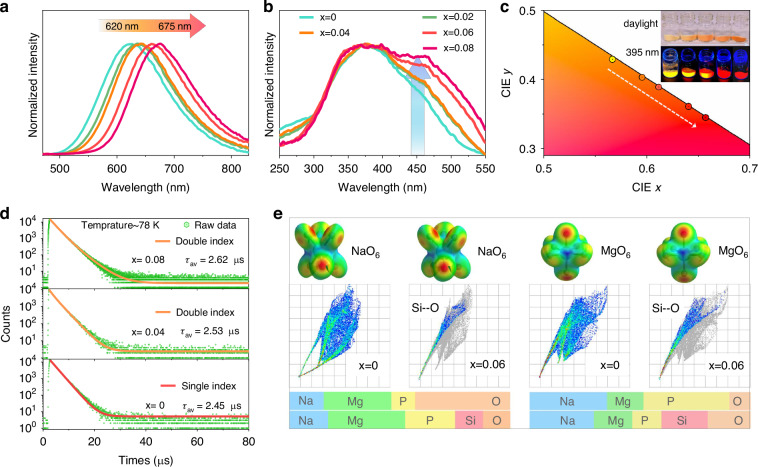
Photoluminescent property of Na_1+x_MgP_1-x_Si_x_O_4_:Eu^2+^ phosphors. **a** Emission and **b** excitation spectra of Na_1+*x*_MgP_1-*x*_Si_*x*_O_4_:Eu^2+^ (*x* = 0–0.08) phosphors. **c** CIE chromaticity coordinates of and the image of Na_1+*x*_MgP_1-*x*_Si_*x*_O_4_:Eu^2+^ phosphors under daylight and 395 nm UV irradiation. **d** Decay curves and fitting results of Na_1+*x*_MgP_1-*x*_Si_*x*_O_4_:Eu^2+^ (*x* = 0, 0.04, 0.08) measured at 78 K under 450 nm pulse laser diode excitation. **e** Hirshfeld surface and 2D finger print plots with d_i_ and d_e_ ranging from 0.6 to 2.4 Å calculated by Crystal Explorer software

The photoluminescence emission (PL) and excitation (PLE) spectra of Na_1+*x*_MgP_1-*x*_Si_*x*_O_4_:Eu (*x* = 0–0.08) are shown in Fig. 2a, b respectively. The as-prepared NaMgPO_4_:Eu phosphor exhibits a wide emission band peaking at 620 nm when excited at 440 nm, attributed to the characteristic 4f^6^5d^1^ → 4f^7^ transition of Eu^2+^. Upon the incorporation of Si ions, the emission peaks shift towards longer wavelengths from 620 to 675 nm (Fig. 2a), accompanied by a gradual enhancement in luminescence intensity, reaching its maximum in the Na_1.06_MgP_0.94_Si_0.06_O_4_:Eu sample (Supplementary Fig. S6). The corresponding PLE spectra for each sample monitored at optimal emission peak contain a broad band from 280 to 550 nm, arising from the 4f → 5d transition of Eu^2+^ (Fig. 2b). It is worth noting that doping Si leads to a progressive enhancement of the excitation intensity in the blue light region, enabling better compatibility with blue LED chips and increasing its potential for commercial use. These alterations observed in PLE and PL spectra lead to a gradual transition of the phosphors’ body color from light yellow to red, as illustrated in the inset of Fig. 2c. In correspondence, the color coordinates derived from the emission spectra of Na_1+*x*_MgP_1-*x*_Si_*x*_O_4_:Eu (*x* = 0–0.08) and the powder’s color under UV light shift from orange to deep-red as Si increases (Fig. 2c).

It is well-known that the luminescent properties of Eu^2+^ are mainly affected by centroid shift, crystal field splitting (CFS) and Stokes shift effects^34^. Among these, the PLE spectrum is mainly related to the spectroscopic polarizability, which in turn is inversely proportional to the square of the electronegativity^35^. The lower electronegativity of Si (1.90) compared to P (2.19) results in the broadening of the PLE spectra in the blue light range upon the substitution of P^5+^→Si^4+^, as depicted in Fig. 2b. With regard to the redshift in PL spectra, which primarily associated with the rise in CFS caused by changes in the local structure of Eu^2+^ ^36^. By comparing the Gaussian peak fitting results of PL spectra at low temperature (78 K) in Supplementary Fig. S7, it is evident that NaMgPO_4_:Eu can be decomposed into a single Gaussian peak, whereas Na_1.06_MgP_0.94_Si_0.06_O_4_:Eu requires two Gaussian peaks for accurate fitting. This indicates that Eu^2+^ ions may occupy only one lattice site in the original sample, while after the introduction of Si, they will occupy two different lattice sites. To validate this viewpoint, we collected decay curves at low temperature (78 K) and analyzed the luminescence dynamic process by fitting them with the mono- and bi-exponential functions, respectively^21^:

1 $$I=\mathop{\sum }\limits_{i=1}^{n}{A}_{i}\exp \left(-\frac{t}{{\tau }_{{\rm{i}}}}\right)$$

where *I* is luminescence intensity, *t* is time, ${\tau }_{i}$ represents lifetime for different components, *n* is index of mono- and bi-exponential functions, and *A*_*i*_ indicates the corresponding fitting constants. The decay curve of NaMgPO_4_:Eu can be well fitted by mono-exponential functions, while the decay trend gradually transitions to a bi-exponential function after Si doping (Fig. 2d). This result further demonstrates the transition from single to double luminescent centers. In addition, the measured decay times at room temperature (300 K) increased from 2.27 to 2.58 μs (Supplementary Fig. S8 and Supplementary Table S5).

Hirshfeld surface (HS) is an emerging method for analyzing the strength of intermolecular interactions and crystal packing^37^. It not only identifies the existing intermolecular interactions but also offers details on the relative surface area associated with each interaction. Here, we primarily focus on analyzing HS with d_e_ that centered around Na and Mg, where d_e_ is defined as the distance from HS to the nearest atom E external to the surface. As illustrated in Fig. 2e and Supplementary Table S6, the introduction of Si led to a gradual increase in the surface area of NaO_6_, while the surface area of MgO_6_ decreased. The Si-O interaction was further examined by 2D fingerprint plots. The surface contribution of Si-O to NaO_6_ and MgO_6_ in Na_1.06_MgP_0.94_Si_0.06_O_4_:Eu is 12.5% and 26.2% respectively. Since the combined contribution of P-O and Si-O to the surface remains nearly constant, it validates that the effective doping of Si will affect the interaction strength of NaO_6_ and MgO_6_.

For a more comprehensive understanding of redshift in PL spectra, we attempt to explore the alterations in the distribution of Eu^2+^ ions pre and post the incorporation of Si atoms. In the NaMgPO_4_ host, there are two different NaO_6_ and MgO_6_ sites, both with coordination number of 6, suitable for Eu^2+^ ion occupation. As Si doping increases, the distortion index of MgO_6_ octahedra remains nearly unchanged, while that of NaO_6_ octahedra gradually decreases (Supplementary Fig. S9a). Due to the positive correlation between CFS and distortion index, the influence of the distortion index on the redshift can be ruled out. Additionally, the average bond length of NaO_6_ polyhedra increases, whereas that of MgO_6_ exhibits a decreasing trend (Supplementary Fig. S9b), which is consistent with the variations in surface area observed in the above HS calculations. Based on the CFS of the 4f^n-1^5d-levels of Ce^3+^ and Eu^2+^ proposed by Dorenbos, the CFS appears to behave as^36^:

2 $${\varepsilon }_{{cf}s}={\beta }_{{poly}}^{Q}{R}_{{av}}^{-2}$$

where ${\beta }_{{poly}}^{Q}$ is a constant that depends on the type of coordination polyhedron, *R*_av_ is the average bond length. It is evident that the CFS is inversely proportional to the square of the bond length, i.e., the occupancy of Eu^2+^ ions in small MgO_6_ octahedra will result in a large CFS. Combining the Gaussian peak and decay curve fitting results, it can be reasonably inferred that Eu^2+^ occupies only the larger NaO_6_ sites in the absence of Si doping (*x* = 0) sample. However, when *x* > 0, Eu^2+^ will enter both NaO_6_ and smaller MgO_6_ sites, leading to the redshift in PL spectra.

### Enhancing external quantum efficiency

**Fig. 3:**
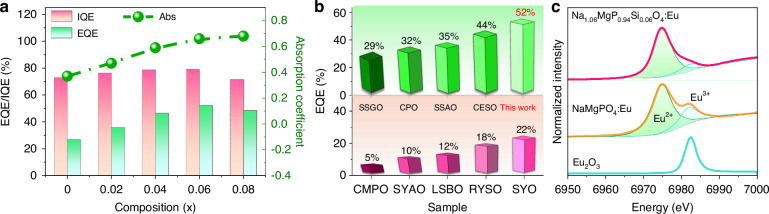
The quantum efficiency of Na_1+x_MgP_1-x_Si_x_O_4_:Eu^2+^ phosphors. **a** The internal/external quantum efficiency and absorption coefficient of Na_1+*x*_MgP_1-*x*_Si_*x*_O_4_:Eu^2+^ phosphors. **b** Comparison of external quantum efficiency of Na_1.06_MgP_0.94_Si_0.06_O_4_:Eu^2+^ with the oxide-based red and deep-red phosphors reported to date, including CsMgPO_4_:Eu (CMPO), Sr_3_YAl_2_O_7.5_:Eu (SYAO), LiSrBO_3_:Eu (LSBO), Rb_3_YSi_2_O_7_:Eu (RYSO), SrY_2_O_4_:Eu (SYO), Sr_2_Sc_0.5_Ga_1.5_O_5_:Eu (SSGO), Ca_4_(PO_4_)_2_O:Eu (CPO), Sr_2_ScAlO_5_:Eu (SSAO) and Ca_1.2_Eu_0.8_SiO_4_ (CESO). **c** Eu L_3_-edge X-ray absorption near-edge structure spectra of the reference sample (Eu_2_O_3_), NaMgPO_4_:Eu^2+^ and Na_1.06_MgP_0.94_Si_0.06_O_4_:Eu^2+^ samples

To assess the commercial viability of the as-synthesized phosphors, their internal quantum efficiency (IQE), external quantum efficiency (EQE), and absorption coefficient (Abs) were measured and are exhibited in Fig. 3a. All samples demonstrate high IQE values exceeding 70%, whereas the initial sample NaMgPO_4_:Eu has an EQE value only of 27% due its weak absorption coefficient (Abs). Fortunately, the Abs values have increased from 0.37 to 0.68 with rising Si content, consistent with the values obtained from the diffuse reflectance spectra (Supplementary Fig. S10a). This leads to a significant improvement in the EQE values of the phosphor series, with the detailed measuring methods for EQE outlined in Supplementary Fig. S11. In particular, compared to the currently developed blue-light-excited red or deep-red oxide phosphors, such as CsMgPO_4_:Eu (CMPO), Sr_3_YAl_2_O_7.5_:Eu (SYAO), LiSrBO_3_:Eu (LSBO), Rb_3_YSi_2_O_7_:Eu (RYSO), SrY_2_O_4_:Eu (SYO), Sr_2_Sc_0.5_Ga_1.5_O_5_:Eu (SSGO), Ca_4_(PO_4_)_2_O:Eu (CPO), Sr_2_ScAlO_5_:Eu (SSAO) and Ca_1.2_Eu_0.8_SiO_4_ (CESO), Na_1.06_MgP_0.94_Si_0.06_O_4_:Eu possesses a record-breaking EQE value of 52% (Fig. 3b)^25,38–45^.

For a deeper insight into the EQE enhancement mechanism, the valence state of Eu was meticulously examined using X-ray absorption near-edge structure (XANES) spectra. Figure 3c shows the normalized Eu-*L*_3_ edge XANES spectra of NaMgPO_4_:Eu and Na_1.06_MgP_0.94_Si_0.06_O_4_:Eu with Eu_2_O_3_ as a reference. Both samples exhibit two peaks at 6974.9 eV and 6982.4 eV, corresponding to the 2p_3/2_ → 5 d transition of Eu^2+^ and Eu^3+^, respectively. It is important to emphasize that the relative intensity of the Eu^3+^ absorption band in Na_1.06_MgP_0.94_Si_0.06_O_4_:Eu experienced a notable decrease, suggesting that a higher Eu^2+^ content effectively enhances the absorption coefficient and EQE values. This is understandable, because as previously mentioned, the introduction of Si not only leads to Eu^2+^ occupying the NaO_6_ sites but also the MgO_6_ sites, consequently amplifying the effective luminescent centers of Eu^2+^. Furthermore, heterovalent doping disrupts the charge distribution within the structure, limiting the ability of Eu^2+^ to occupy Na^+^ sites. The incorporation of low-valence Si^4+^ can facilitate the formation of the equivalent co-substitution Na^+^ + P^5+^$\leftrightarrow$Eu^2+^ + Si^4+^, aiding in restoring a balanced charge state and thereby enhancing the presence of Eu^2+^.

### Enhancing thermal stability and exceptional water stability

**Fig. 4:**
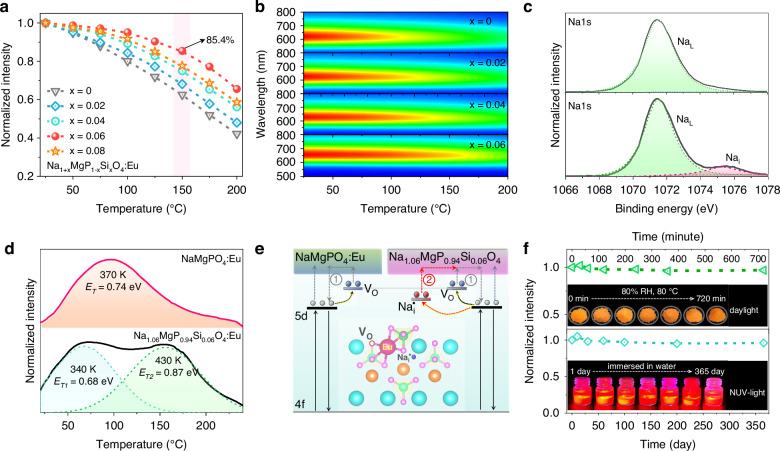
Stability of synthesized phosphors to heat and water. **a** Normalized emission intensities and **b** temperature dependent emission spectra of Na_1+*x*_MgP_1-*x*_Si_*x*_O_4_:Eu^2+^ (*x* = 0–0.08) as a function of temperature in the range of 20–200 °C. **c** Na1s XPS spectra of NaMgPO_4_:Eu^2+^ (up) and Na_1.06_MgP_0.94_Si_0.06_O_4_:Eu^2+^ (down). **d** Thermoluminescence spectra of NaMgPO_4_:Eu^2+^ and Na_1.06_MgP_0.94_Si_0.06_O_4_:Eu^2+^ in the temperature range of 25–200 °C. **e** Schematic illustration of the thermal quenching process mechanism in NaMgPO_4_:Eu^2+^ and Na_1.06_MgP_0.94_Si_0.06_O_4_:Eu^2+^. **f** Water-stability of Na_1.06_MgP_0.94_Si_0.06_O_4_:Eu^2+^ powder

Another crucial characteristic for an excellent phosphor is its resistance to thermal quenching (TQ). Figure 4a presents the temperature-dependent integrated emission intensity of Na_1+*x*_MgP_1-*x*_Si_*x*_O_4_:Eu (*x* = 0–0.08) within the temperature range of 25–200 °C. Due to the TQ effect, the PL intensity of all samples diminishes with rising temperatures. In contrast to NaMgPO_4_:Eu, the thermal stability of the Si-doped samples exhibits a gradual enhancement with the increase in *x*, reaching its peak at *x* = 0.06. Specifically, the most thermally stable phosphor, Na_1.06_MgP_0.94_Si_0.06_O_4_:Eu, retains 85.4% of its initial intensity at room temperature when subjected to 150 °C. The two-dimensional temperature-dependent PL spectra in Fig. 4b offers a more intuitive illustration of the enhanced thermal stability achieved through Si doping. The high thermal stability of Na_1.06_MgP_0.94_Si_0.06_O_4_:Eu provides assurance for its application in high-power light sources.

According to the thermal ionization model proposed by Dorenbos, thermal stability is primarily dictated by the energy barrier Δ*E* between the conduction band and the 5 *d* energy level of Eu^2+^ ions^46^. Based on the UV-visible diffuse reflectance spectra and Kubelka-Munk function analysis, the Tauc plot in Supplementary Fig. S10c reveals a decrease in the band gap of the host material from 5.16 to 5.04 eV as the *x* value increases from 0 to 0.06^47^. Additionally, DFT calculations further confirmed a decreasing trend in the band gap, from 5.16 to 4.56 eV, as shown in Supplementary Fig. S12. This is inconsistent with the improvement in thermal stability shown in Fig. 4a, as a smaller Δ*E* implies a higher TQ effect. Hence, the recently proposed model for enhancing thermal stability through defect energy levels should be taken into consideration to elucidate TQ^22,48^.

Based on the refined results and DFT calculations above, the substitution of Si^4+^ for P^5+^ may introduce interstitial Na, resulting in the formation of interstitial defects ${{\rm{Na}}}_{{\rm{i}}}^{\bullet }$, while the high-temperature reducing synthesis environment can lead to the formation of oxygen vacancies V_O_. Consequently, the Na1s and O1s XPS spectra of NaMgPO_4_:Eu and Na_1.06_MgP_0.94_Si_0.06_O_4_:Eu were measured and analyzed in Fig. 4c and Supplementary Fig. S13. The binding energy of 1072 eV corresponds to lattice Na (Na_L_), while the peak at 1075 eV in Na_1.06_MgP_0.94_Si_0.06_O_4_:Eu is attributed to contributions from interstitial Na defects (${{\rm{Na}}}_{{\rm{i}}}^{\bullet }$)^49^.There are three fitted peaks present in both samples, among which the middle peaks are ascribed to the oxygen vacancy (Supplementary Fig. S13)^50^. The changes in the thermoluminescence spectra offer further support for this hypothesis. As illustrated in Fig. 4d, the TL spectrum of Na_1.06_MgP_0.94_Si_0.06_O_4_:Eu exhibits a significant broadening, with two characteristic trap depths (*E*_T_) estimated at 0.68 and 0.87 eV using the crude relationship *E*_*T*_ = *T/*500 eV. Compared with the shallow trap (V_O_ ~ 0.74 eV) in NaMgPO_4_:Eu, the introduction of Si created extra deeper defect levels (${{\rm{Na}}}_{{\rm{i}}}^{\bullet }$ ~ 0.87 eV). Based on the above analysis, Fig. 4e depicts a simplified schematic to elucidate the mechanism of the enhancing thermal stability. As temperature increases, partial electrons transfer to the conduction band (CB), initiating the TQ process. For NaMgPO_4_:Eu, some excited electrons will be captured by oxygen defect levels (V_O_) and subsequently return from the CB to the 5 d level (process (1)) with the assistance of thermal vibration. However, the compensatory impact on TQ is constrained due to the shallow defect energy level of V_O_. In contrast, the emergence of additional deeper defect levels ${{\rm{Na}}}_{{\rm{i}}}^{\bullet }$ in Na_1.06_MgP_0.94_Si_0.06_O_4_:Eu continues to mitigate the TQ effect, thereby bolstering the material’s thermal stability.

The moisture stability of phosphors is equally vital when evaluating their potential for commercial use. To assess the robustness of Na_1.06_MgP_0.94_Si_0.06_O_4_:Eu under extreme environmental conditions, the samples were exposed to 80% relative humidity and 80 °C for 0-720 min, respectively. Remarkably, despite the harsh conditions, the emission intensity remained at 96% of the initial value after the exposure (Fig. 4f and Supplementary Fig. S14b), and the color of the powders remained almost unchanged. Furthermore, even after submerging the samples in water for 365 days (Supplementary Fig. S14a), the intensity remained above 95%. These results clearly demonstrate the outstanding water stability of Na_1.06_MgP_0.94_Si_0.06_O_4_:Eu phosphor.

### Fabrication and performance of LEDs

**Fig. 5:**
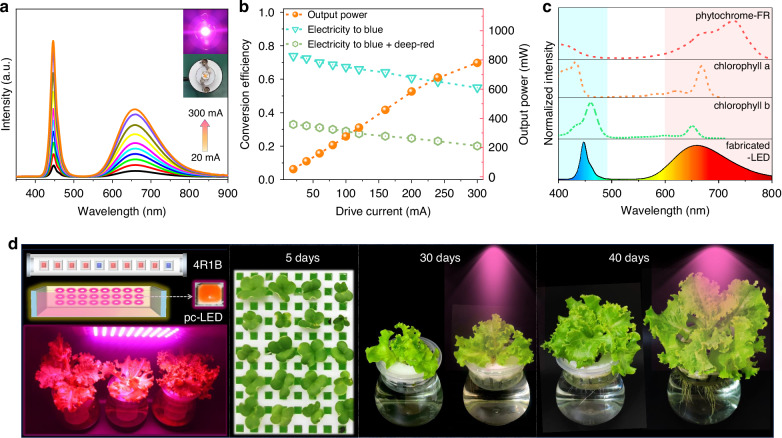
Performance and application of fabricated pc-LEDs. **a** Electroluminescent (EL) spectra and photographs of the LED fabricated using a GaN chip (λ_max_ = 440 nm) and Na_1.06_MgP_0.94_Si_0.06_O_4_:Eu^2+^ phosphor encapsulation under current of 20-300 mA. **b** Output power, electricity-to-blue light and electricity-to-purple light conversion efficiency under various drive current. **c** Comparation of the EL spectra of fabricated LED with absorption spectra of chlorophyll-a, chlorophyll-b and phytochrome-FR in plants. **d** Schematic diagram of commercial 4R1B plant growth lighting device and the fabricated pc-LED, as well as photos of lettuce cultivation under various lighting conditions

To demonstrate the feasibility of the material in plant lighting, LED devices were prepared using Na_1.06_MgP_0.94_Si_0.06_O_4_:Eu and blue GaN (440 nm) chips for encapsulation. The intensities of the blue and deep-red emission bands exhibit a continuous increase with increasing current, without any observed saturation phenomenon in the range of 20–300 mA (Fig. 5a). Figure 5b depicts the output powers and corresponding photoelectric efficiencies of the manufactured LED at different operating currents. As the driving current increases, the output power gradually rises, reaching a high of 780 mW at 300 mA. However, the electricity to blue and deep-red efficiency gradually diminishes, attributed to the “efficiency droop” phenomenon of the GaN blue chip. Figure 5c compares the absorption spectra of crucial chlorophyll a, chlorophyll b and phytochrome-FR for plant photosynthesis with the emission spectra of the LED devices. It is evident that the broad emission band and appropriate peak position of the Na_1.06_MgP_0.94_Si_0.06_O_4_:Eu allow the spectra of the fabricated LED to cover nearly all the absorption regions of plant phytochromes. These findings suggest that Na_1.06_MgP_0.94_Si_0.06_O_4_:Eu can be regarded as a promising luminescent material for plant lighting.

To visually evaluate the effects of fabricated LEDs on indoor plant growth, a high-power plant illumination device with an output power of approximately 20 W was constructed by serially connecting fifty 1 W LED units (Fig. 5d). Initially, the lettuce seeds were soaked in a plant nutrient solution and incubated for five days to facilitate seedling growth. Subsequently, the seedlings were divided into two groups for a control experiment, with one group being cultivated under natural light, and the other group receiving additional artificial LED radiation during the evening. In order to ensure the accuracy of the experimental outcomes, we maintain consistency in the concentration and volume of the nutrient solution utilized for both groups. The image depicted in Fig. 5d illustrates that following a 40-day cultivation period, the lettuce exposed to supplemental LED light displayed a distinct growth advantage. This further confirms that artificial LED lighting effectively complements the essential absorption light of phytochromes, thereby promoting plant growth, regardless of the indoor lighting conditions. Therefore, the deep-red phosphor Na_1.06_MgP_0.94_Si_0.06_O_4_:Eu holds promising prospects for applications in indoor cultivation.

## Discussion

In conclusion, we have successfully synthesized a phase stable olivine Na_1.06_MgP_0.94_Si_0.06_O_4_:Eu deep-red phosphor through compositional engineering, showcasing high EQE and excellent thermal stability. The PL spectra of composition-exchanged phosphors exhibited a significant redshift from the pristine red to the deep-red region due to the replacement of P^5+^ ions with Si^4+^ ions. Simultaneously, the excitation intensity in the blue light region is notably amplified, enabling better compatibility with blue GaN chips. Furthermore, the introduction of Si not only increases the number of Eu^2+^ luminescent centers, boosting EQE from 27% to 52%, but also introduces additional deep defect levels, improving thermal stability from 62.5% to 85.4%. In addition, Na_1.06_MgP_0.94_Si_0.06_O_4_:Eu demonstrates outstanding water stability, retaining more than 95% of its original luminescent intensity even after submersion in water for one year. The performance of encapsulated LEDs and the comparison of lettuce growth trends suggest that Na_1.06_MgP_0.94_Si_0.06_O_4_:Eu can be considered an alternative phosphor for plant lighting.

## Materials and methods

### Sample preparation

The traditional solid-state reaction synthesis is conducted using Na_2_CO_3_ (99.9%), MgO (99.9%), NH_4_H_2_PO_4_ (99.9%), SiO_2_ (99.9%), and Eu_2_O_3_ (99.99%) as raw materials. The stoichiometric mixtures were thoroughly grounded and transferred into an alumina crucible and pre-sintered at 600 °C for 4 h in ambient air to decompose the ammonium and carbonate components. They were then re-ground and sintered individually at 1230 °C, 1250 °C, 1280 °C, 1290 °C, and 1300 °C for 6 h in a forming gas atmosphere (N_2_:H_2_ = 80:20) to produce samples of NaMgPO_4_:Eu, Na_1.02_MgP_0.98_Si_0.02_O_4_:Eu, Na_1.04_MgP_0.96_Si_0.04_O_4_:Eu, Na_1.06_MgP_0.94_Si_0.06_O_4_:Eu and Na_1.08_MgP_0.92_Si_0.08_O_4_:Eu samples, respectively. The Eu doping concentration for all samples is 2.5 mol%. The high-purity NaMgPO_4_:Eu sample for Rietveld refinement was obtained using the rapid annealing method, i.e. the samples were instantly removed from the high-temperature box furnace under a carbon-reducing atmosphere after sintering at 1230 °C for 6 h achieve the purpose of instant annealing.

### Characterization

The powder XRD patterns were all checked in range of 2θ = 5°–120° for phase purity using a D8 Advance diffractometer (Bruker Corporation, Germany) operating at 40 kV and 40 mA with Cu K*a* radiation (λ = 1.5405 Å). The crystal structure was refined based on the Rietveld method using TOPAS 4.2 software. The sample morphologies were analyzed using a scanning electron microscope (SEM, JEOL JSM-6510A) equipped with an energy dispersive spectroscopy (EDS) analyzer. Eu L3-edge XANES and EXAFS data were recorded on the beamline at the Beijing Synchrotron Radiation Facility (1W1B). The photoluminescence (PL) and photoluminescence excitation spectra (PLE) were monitored using a spectrometer (FLS 1000, Edinburgh) equipped with a 450 W xenon (Xe) lamp and a temperature control instrument. Diffuse reflectance spectra were recorded on a Hitachi UH4150 ultraviolet-visible-near infrared spectrophotometer. The room temperature (300 K) and low temperature (80 K) decay curves were measured using an FLS1000 instrument equipped with a 450 nm pulse laser diode as the excitation source, and liquid nitrogen was utilized to cool the temperature. The quantum efficiency (QE) was measured using the FLS1000 attached a barium sulfate coated integrating sphere. Thermoluminescence (TL) spectra were monitored by a TOSL-3DS instrument (Ai-di-rui-sheng Company, China) equipped with UV radiation source. X-ray photoelectron spectroscopy (XPS) spectra were measured at room temperature using an VG ESCALABMK II electron spectrometer with a Mg K*α* (1200 eV) line.

### Fabrication of pc-LED

Phosphor converted LED (pc-LED) devices were fabricated by integrating the Na_1.06_MgP_0.94_Si_0.06_O_4_:Eu on an GaN LED chip (λ_max_ = 440 nm). The devices were encapsulated in a phosphor/silicone resin mixture, and then cured in an oven at 120 °C for 2 h to form the final pc-LEDs devices. Photoelectric properties of the as-fabricated pc-LEDs were measured using auto-temperature LED opto-electronic analyser (ATA-1000, Everfine, China).

### Plant cultivation

Plant seeds are firstly germinated and grown into seedlings using plant nutrient solution. Afterward, the seedlings are divided into two groups, a control group (referred to as CG) and an experimental group (referred to as EG). Both groups of lettuce receive the same sunlight during the day, but at night the control group is placed in a dark environment, while the experimental group is exposed to deep-red light from the fabricated LEDs for 12 h. Cultivate under the above specified conditions for 40 days and observe the growth trend changes of two groups of lettuce.

### Calculations

To investigate the structural properties, we carried out all periodic DFT calculations of NaMgPO_4_ based on the density functional theory (DFT)^51,52^. Supercell of NaMgPO_4_ were established to replace P to Si or add Na in the vacancy of the lattice. The generalized gradient approximation (GGA) with the Perdew-Burke-Ernzerhof (PBE) functional was employed as the exchange-correlation potential. The cut-off energy E_cut_ of 500 eV was used throughout all the calculations. A gamma-centered 3 × 3 × 3 k-mesh grid in the Brillouin zone was applied to determine the self-consistent charge density using Monkhorst-Packscheme. The crystal lattice was fully relaxed until the atomic force was less than 0.02 eV/Å. The energy convergence criterion for self-consistent electronic calculation was set to 10^−5 ^eV/atom.

To investigate the effects of external atoms on the NaO_6_ and MgO_6_ polyhedra, calculations of the Hirshfeld surfaces were conducted using Crystal Explorer software. Crystal Explorer is a crystal visualization software based on quantum mechanical theory for the study of intermolecular interactions and stacking in crystalline materials by Hirshfeld surface analysis. The selection of the corresponding surfaces enables accurate and efficient calculations of the energy of intermolecular interactions and energy frames. By mapping these properties, as well as other distance- and curvature-dependent metrics on Hirshfeld surfaces, Crystal Explorer provides unique insights into the intracrystalline environment.

## Supplementary information

Supplementary Information

## References

[1] Van Delden, S. H. et al. Current status and future challenges in implementing and upscaling vertical farming systems. *Nat. Food* **2**, 944–956 (2021). 37118238 10.1038/s43016-021-00402-w

[2] Chowdhury, M. et al. Effects of temperature, relative humidity, and carbon dioxide concentration on growth and glucosinolate content of kale grown in a plant factory. *Foods* **10**, 1524 (2021). 34359392 10.3390/foods10071524 PMC8306225

[3] Olle, M. & Viršile, A. The effects of light-emitting diode lighting on greenhouse plant growth and quality. *Agric. Food Sci.* **22**, 223–234 (2013).

[4] He, R. et al. UV-A and FR irradiation improves growth and nutritional properties of lettuce grown in an artificial light plant factory. *Food Chem.* **345**, 128727 (2021). 33307433 10.1016/j.foodchem.2020.128727

[5] Liu, G. C. et al. Laser-driven broadband near-infrared light source with watt-level output. *Nat. Photonics* **18**, 562–568 (2024).

[6] Kang, W. H. et al. Quantification of spectral perception of plants with light absorption of photoreceptors. *Plants* **9**, 556 (2020). 32349252 10.3390/plants9050556 PMC7285096

[7] Budagovsky, A. V. et al. Influence of far-red light coherence on the functional state of plants. *Phys. Rev. E* **103**, 012411 (2021). 33601635 10.1103/PhysRevE.103.012411

[8] Huang, W. T. et al. Plant growth modeling and response from broadband phosphor-converted lighting for indoor agriculture. *ACS Appl. Mater. Interfaces* **15**, 32589–32596 (2023). 37364173 10.1021/acsami.3c06454

[9] Fang, S. Q. et al. Light keys open locks of plant photoresponses: A review of phosphors for plant cultivation LEDs. *J. Alloy. Compd.* **902**, 163825 (2022).

[10] Hu, T. et al. Glass crystallization making red phosphor for high-power warm white lighting. *Light Sci. Appl.* **10**, 56 (2021). 33712554 10.1038/s41377-021-00498-6 PMC7955133

[11] Liang, S. S. et al. A highly efficient red emitting phosphor with enhanced blue-light absorption through a local crystal field regulation strategy. *Chem. Eng. J.* **429**, 132231 (2022).

[12] Wang, S. C. et al. Tremendous acceleration of plant growth by applying a new sunlight converter Sr_4_Al_14-*x*_Ga_*x*_O_25_: Mn^4+^ breaking parity forbidden transition. *Adv. Sci.* **10**, 2204418 (2023). 10.1002/advs.202204418 PMC9839862 36424134

[13] Kang, X. J. et al. A novel blue-light excitable Pr^3+^ doped (Sr, Ba)LaMgTaO_6_ phosphor for plant growth lighting. *J. Rare Earths* **41**, 666–672 (2023).

[14] Lu, Z. Z. et al. Double perovskite Ba_2_LaNbO_6_: Mn^4+^, Yb^3+^ phosphors: potential application to plant-cultivation LEDs. *Dyes Pigments* **160**, 395–402 (2019).

[15] Jia, Z. W. et al. Strategies to approach high performance in Cr^3+^-doped phosphors for high-power NIR-LED light sources. *Light Sci. Appl.* **9**, 86 (2020). 32435469 10.1038/s41377-020-0326-8 PMC7229223

[16] Yang, C. W. et al. Highly-efficiency far-red emission in Cr^3+^ activated Ca_1.8_Mg_1.2_Al_2_Ge_3_O_12_ toward plant precise lighting. *Adv. Optical Mater.* **12**, 2303235 (2024).

[17] He, F. Q. et al. A general ammonium salt assisted synthesis strategy for Cr^3+^-doped hexafluorides with highly efficient near infrared emissions. *Adv. Funct. Mater.* **31**, 2103743 (2021).

[18] Dai, X. Y. et al. Novel Cr^3+^-doped garnet phosphor with broadband efficient far-red emission for photochrome matching plant-lighting. *Adv. Optical Mater.* **12**, 2302380 (2024).

[19] Yang, C. W. et al. Towards promoting plant growth and fruit maturation: a highly efficient and thermally stable Cr^3+^ doped far-red phosphor. *J. Mater. Chem. C.* **12**, 12451–12457 (2024).

[20] Wang, Z. B. et al. Mining unexplored chemistries for phosphors for high-color-quality white-light-emitting diodes. *Joule* **2**, 914–926 (2018).

[21] Qiao, J. W. et al. Divalent europium-doped near-infrared-emitting phosphor for light-emitting diodes. *Nat. Commun.* **10**, 5267 (2019). 31748595 10.1038/s41467-019-13293-0 PMC6868216

[22] Kim, Y. H. et al. A zero-thermal-quenching phosphor. *Nat. Mater.* **16**, 543–550 (2017). 28191898 10.1038/nmat4843

[23] Tsai, Y. T. et al. Structural ordering and charge variation induced by cation substitution in (Sr, Ca)AlSiN_3_: Eu phosphor. *J. Am. Chem. Soc.* **137**, 8936–8939 (2015). 26161898 10.1021/jacs.5b06080

[24] Pust, P. et al. Narrow-band red-emitting Sr[LiAl_3_N_4_]: Eu^2+^ as a next-generation LED-phosphor material. *Nat. Mater.* **13**, 891–896 (2014). 24952748 10.1038/nmat4012

[25] Sato, Y. et al. Tailoring of deep-red luminescence in Ca_2_SiO_4_: Eu^2+^. *Angew. Chem. Int. Ed.* **53**, 7756–7759 (2014). 10.1002/anie.201402520 24916117

[26] Hasegawa, T. et al. Phase stabilization of red-emitting olivine-type NaMgPO_4_: Eu^2+^ phosphors *via* molten-phase quenching. *Inorg. Chem. Front.* **7**, 4040–4051 (2020).

[27] Kim, S. W. et al. Efficient red emission of blue-light excitable new structure type NaMgPO_4_: Eu^2+^ phosphor. *ECS Solid State Lett.* **2**, R49–R51 (2013).

[28] Du, Y. Z. et al. Advanced effect of the substitution of Zn^2+^ on the solid-state synthesis of red phosphor, high temperature phase NaMgPO_4_: Eu^2+^. *Adv. Optical Mater.* **12**, 2302183 (2024).

[29] Zhang, Z. & Tang, W. J. Efficient sensitization of Eu^2+^/Mn^2+^ emissions by Ce^3+^ doping in NaMgPO_4_ host under UV excitation. *Appl. Phys. A* **122**, 229 (2016).

[30] Guin, M., Tietz, F. & Guillon, O. New promising NASICON material as solid electrolyte for sodium-ion batteries: Correlation between composition, crystal structure and ionic conductivity of Na_3+x_Sc_2_Si_x_P_3−x_O_12_. *Solid State Ion.* **293**, 18–26 (2016).

[31] Kim, D. et al. Highly luminous and thermally stable mg-substituted Ca_2-*x*_Mg_*x*_SiO_4_: Ce (0 ≤ *x* ≤ 1) phosphor for NUV-LEDs. *Inorg. Chem.* **56**, 12116–12128 (2017). 28949134 10.1021/acs.inorgchem.7b01166

[32] Boughzala, K. et al. Spectroscopic studies and Rietveld refinement of strontium-britholites. *J. Rare Earths* **26**, 483–489 (2008).

[33] Kim, D. et al. Highly luminous Ba_2_SiO_4-δ_N_2/3δ_: Eu^2+^ phosphor for NUV-LEDs: origin of PL-enhancement by N^3−^-substitution. *Materials* **13**, 1859 (2020). 32326554 10.3390/ma13081859 PMC7215923

[34] Qin, X. et al. Lanthanide-activated phosphors based on 4f-5d optical transitions: theoretical and experimental aspects. *Chem. Rev.* **117**, 4488–4527 (2017). 28240879 10.1021/acs.chemrev.6b00691

[35] Dorenbos, P. Ce^3+^ 5d-centroid shift and vacuum referred 4f-electron binding energies of all lanthanide impurities in 150 different compounds. *J. Lumin.* **135**, 93–104 (2013).

[36] Wang, S. X. et al. Crystal field splitting of 4f^n−1^5d-levels of Ce^3+^ and Eu^2+^ in nitride compounds. *J. Lumin.* **194**, 461–466 (2018).

[37] Spackman, P. R. et al. *CrystalExplorer*: a program for Hirshfeld surface analysis, visualization and quantitative analysis of molecular crystals. *J. Appl. Crystallogr.* **54**, 1006–1011 (2021). 34188619 10.1107/S1600576721002910 PMC8202033

[38] Huang, Y. L. & Seo, H. J. Luminescence of Eu^2+^ ions in CsMgPO_4_ phosphor: anomalous emission and its origin. *J. Electrochem. Soc.* **158**, J260–J263 (2011).

[39] Hu, T. et al. Eu^2+^ stabilized at octahedrally coordinated Ln^3+^ site enabling red emission in Sr_3_LnAl_2_O_7.5_ (Ln = Y or Lu) phosphors. *Adv. Optical Mater.* **9**, 2100077 (2021).

[40] Zhang, J. L. et al. LiSrBO_3_: Eu^2+^: A novel broad-band red phosphor under the excitation of a blue light. *Mater. Lett.* **79**, 100–102 (2012).

[41] Qiao, J. W. et al. Site-selective occupancy of Eu^2+^ toward blue-light-excited red emission in a Rb_3_YSi_2_O_7_: Eu phosphor. *Angew. Chem. Int. Ed.* **58**, 11521–11526 (2019). 10.1002/anie.201905787 31167043

[42] Yang, Z. Y. et al. Giant red-shifted emission in (Sr, Ba)Y_2_O_4_: Eu^2+^ phosphor toward broadband near-infrared luminescence. *Adv. Funct. Mater.* **32**, 2103927 (2022).

[43] Yang, Z. Y. et al. Rapid synthesis of red-emitting Sr_2_Sc_0.5_Ga_1.5_O_5_: Eu^2+^ phosphors and the tunable photoluminescence via Sr/Ba substitution. *Adv. Optical Mater.* **9**, 2100131 (2021).

[44] Deng, D. G. et al. Ca_4_(PO_4_)_2_O: Eu^2+^ red-emitting phosphor for solid-state lighting: structure, luminescent properties and white light emitting diode application. *J. Mater. Chem. C.* **1**, 3194–3199 (2013).

[45] Zhou, T. L. et al. A red oxide phosphor, Sr_2_ScAlO_5_: Eu^2+^ with perovskite-type structure, for white light-emitting diodes. *Chin. Phys. B* **19**, 127808 (2010).

[46] Lin, Y. C., Bettinelli, M. & Karlsson, M. Unraveling the mechanisms of thermal quenching of luminescence in Ce^3+^-doped garnet phosphors. *Chem. Mater.* **31**, 3851–3862 (2019).

[47] Qiao, J. W. et al. Near-infrared light-emitting diodes utilizing a europium-activated calcium oxide phosphor with external quantum efficiency of up to 54.7. *Adv. Mater.* **34**, 2201887 (2022). 10.1002/adma.202201887 35426472

[48] Qiao, J. W. et al. Eu^2+^ site preferences in the mixed cation K_2_BaCa(PO_4_)_2_ and thermally stable luminescence. *J. Am. Chem. Soc.* **140**, 9730–9736 (2018). 29985612 10.1021/jacs.8b06021

[49] Citrin, P. H., Wertheim, G. K. & Baer, Y. Many-body processes in x-ray photoemission line shapes from Li, Na, Mg, and Al metals. *Phys. Rev. B* **16**, 4256–4282 (1977).

[50] Du, M. et al. Oxygen-vacancy and phosphate coordination triggered strain engineering of vanadium oxide for high-performance aqueous zinc ion storage. *Nano Energy* **89**, 106477 (2021).

[51] Kresse, G. & Furthmuller, J. Efficient iterative schemes for ab initio total-energy calculations using a plane-wave basis set. *Phys. Rev. B* **54**, 11169–11186 (1996). 10.1103/physrevb.54.11169 9984901

[52] Kresse, G. & Furthmüller, J. Efficiency of ab-initio total energy calculations for metals and semiconductors using a plane-wave basis set. *Comput. Mater. Sci.* **6**, 15–50 (1996). 10.1103/physrevb.54.11169 9984901

