# Supplementary material for: Compositional engineering of phase-stable and highly efficient deep-red emitting phosphor for advanced plant lighting systems
Source: Light Sci Appl. 2024 Dec 11;13:319. doi: 10.1038/s41377-024-01679-9 (PMC11634966; doi:10.1038/s41377-024-01679-9)
Supplement: Supplementary file 1 — Supplementary Information [file 41377_2024_1679_MOESM1_ESM.docx]

**Compositional engineering of phase-stable and highly efficient deep-red emitting phosphor for advanced plant lighting systems**

Jianwei Qiao^1*^, Dehong Li^1^, Qiufeng Shi^1*^, Haijie Guo^1^, Ping Huang^1^, Lei Wang^1^^*^

^1^ College of Physics and Optoelectronic Engineering, Taiyuan University of Technology, Taiyuan 030024, China

**Correspondence:**

Jianwei Qiao (qiaojianwei@tyut.edu.cn)

Qiufeng Shi (shiqiufeng@tyut.edu.cn)

Lei Wang (wanglei_keke@163.com)


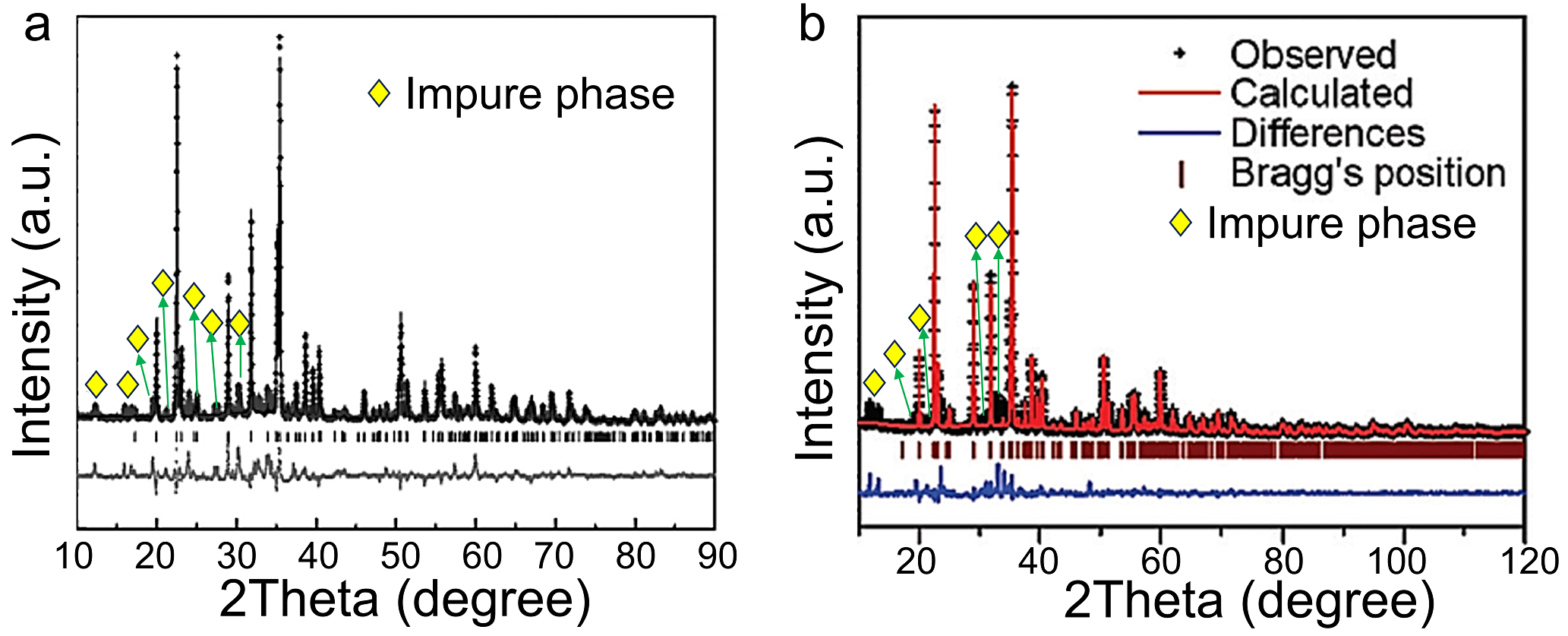


**Fig. S1** Rietveld refinement results of O-NaMgPO_4_ XRD patern synthesized through arc-imaging furnace **(a)** and rapid quenching method **(b)** reported by Toda et al.^1, 2^ Obviously, there are impure phases in the samples using both synthesis methods.


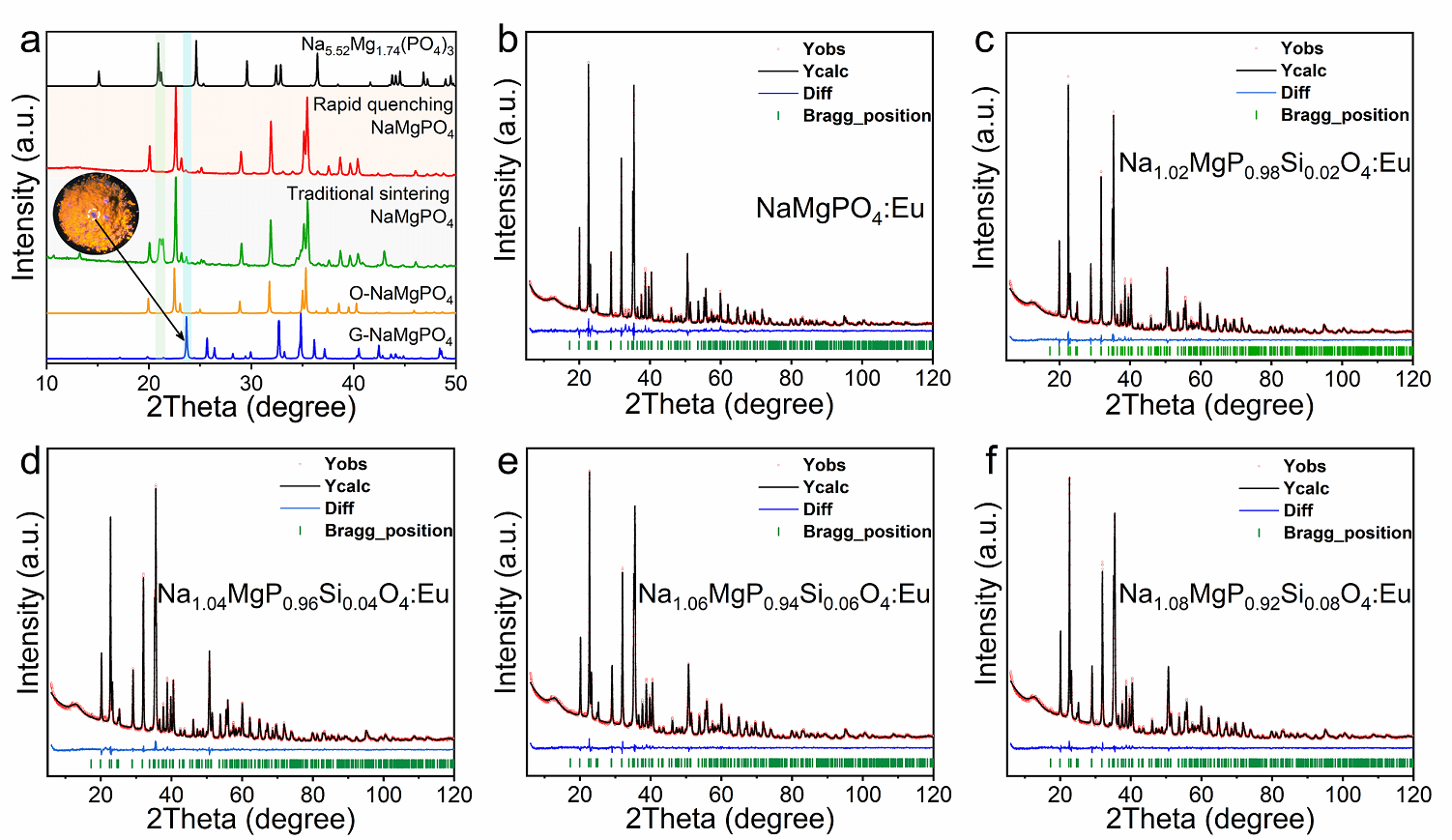


**Fig. S2 (a)** XRD patterns of NaMgPO_4_:Eu synthesized by traditional high-temperature solid state and rapid quenching method, and the simulated standard card of O-NaMgPO_4_, G-NaMgPO_4_, and Na_5.52_Mg_1.4_(PO_4_)_3_. **(b-f)** Rietveld refinement results of Na_1+_*_x_*MgP_1-_*_x_*Si*_x_*O_4_:Eu^2+^ (*x* = 0-0.08) X-ray powder diffraction pattern.


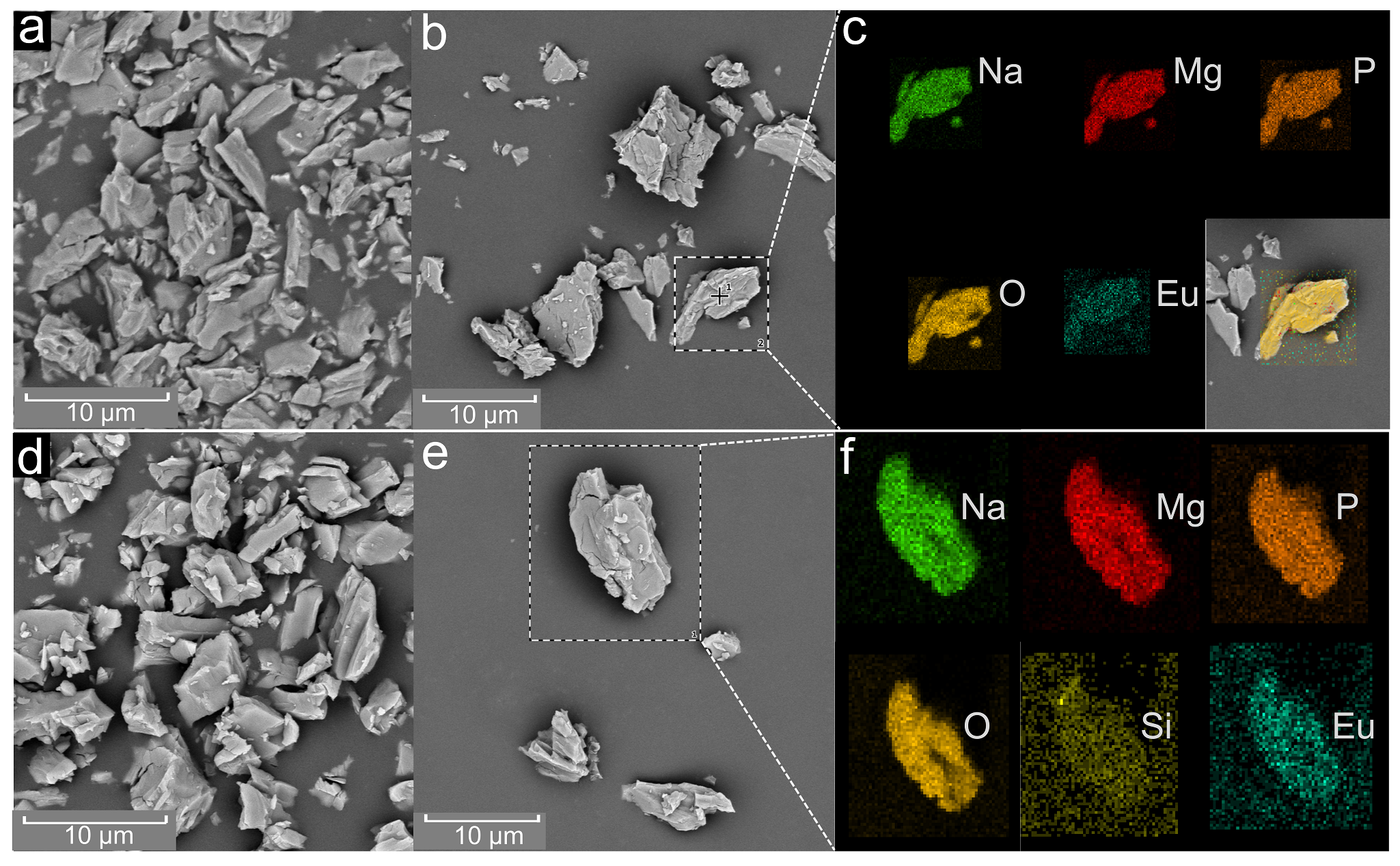


**Fig. S3** **(a)** SEM images of the NaMgPO_4_:Eu microcrystal particles and the enlarged particles **(b)**. **(c)** EDS elemental mapping of the cross section of NaMgPO_4_:Eu. **(d)** SEM images of the Na_1.06_MgP_0.94_Si_0.06_O_4_:Eu microcrystal particles and the enlarged particles **(e)**. **(f)** EDS elemental mapping of the cross section of Na_1.06_MgP_0.94_Si_0.06_O_4_:Eu.


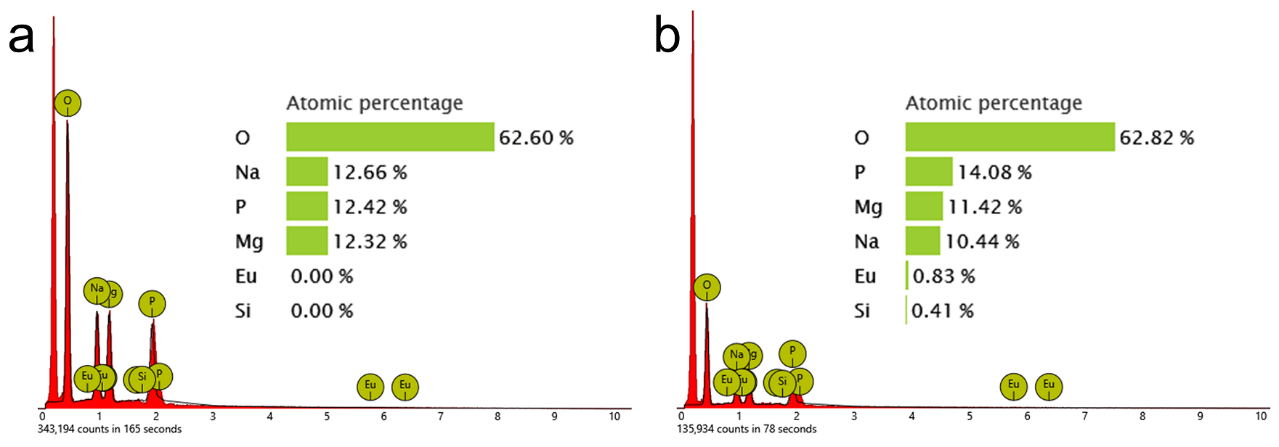


**Fig. S4** EDX spectra of NaMgPO_4_:Eu **(a)** and Na_1.06_MgP_0.94_Si_0.06_O_4_:Eu **(b)**. The detected mole ratio of Na, Mg, P elements is close to the stoichiometric ratio of NaMgPO_4_.


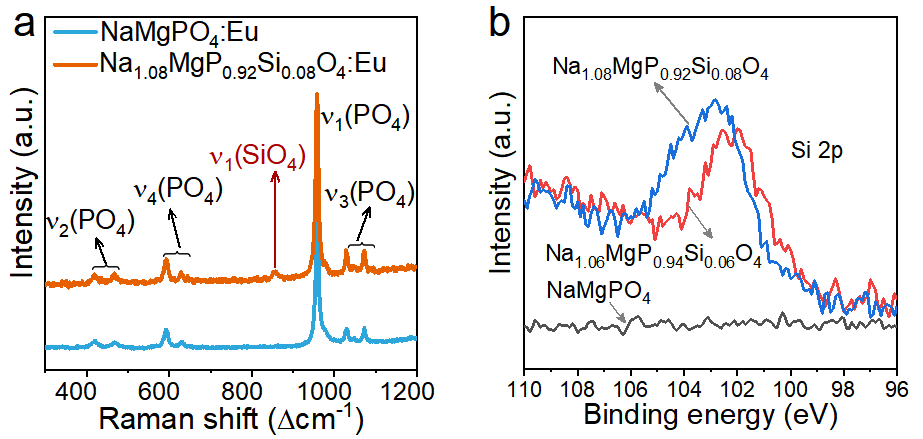


**Fig. S5** (a) Raman spectra of NaMgPO_4_:Eu and Na_1.06_MgP_0.94_Si_0.06_O_4_:Eu samples. (b) Si-2p XPS binding energies of NaMgPO_4_:Eu, Na_1.06_MgP_0.94_Si_0.06_O_4_:Eu and Na_1.08_MgP_0.92_Si_0.08_O_4_:Eu.


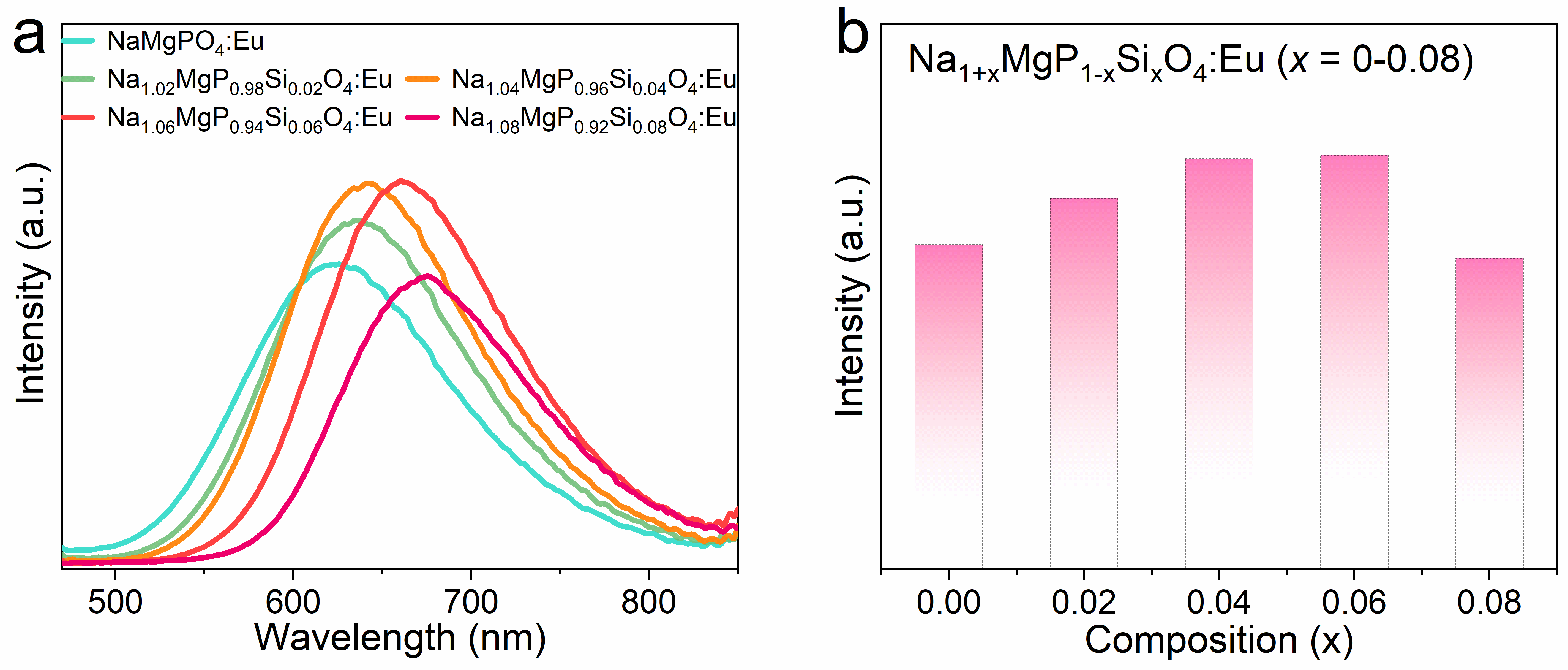


**Fig. S6** Emission spectra **(a)** and integration intensity of Na_1+_*_x_*MgP_1-_*_x_*Si*_x_*O_4_:Eu^2+^ (*x* = 0-0.08) phosphors **(b)**.


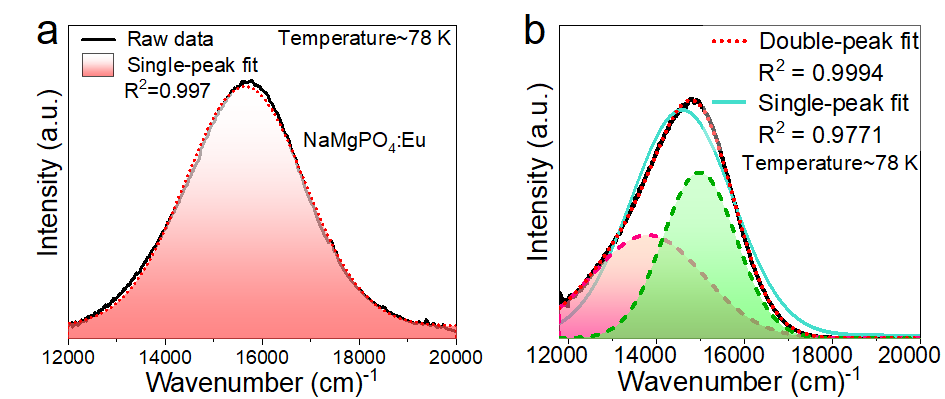


**Fig. S7** The emission spectrum of the NaMgPO_4_:Eu^2+^ measured at low temperature 78 K and Gaussian fitting curves.


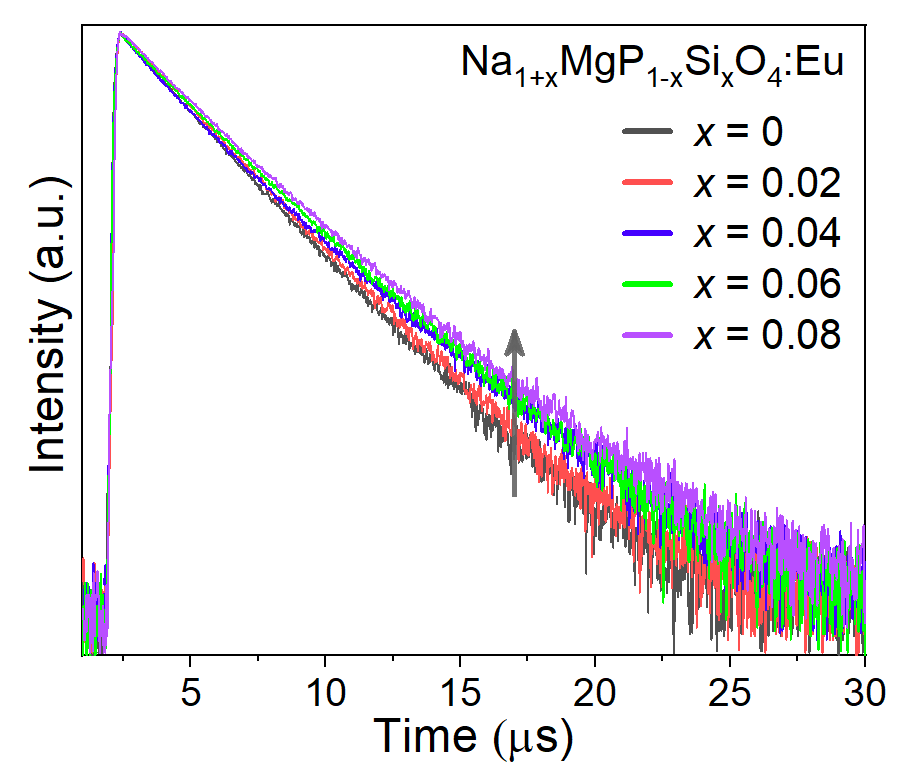


**Fig. S8** Decay curves of Na_1+_*_x_*MgP_1-_*_x_*Si*_x_*O_4_:Eu^2+^ (*x* = 0-0.08) measured at 300 K under 450 nm pulse laser diode excitation.


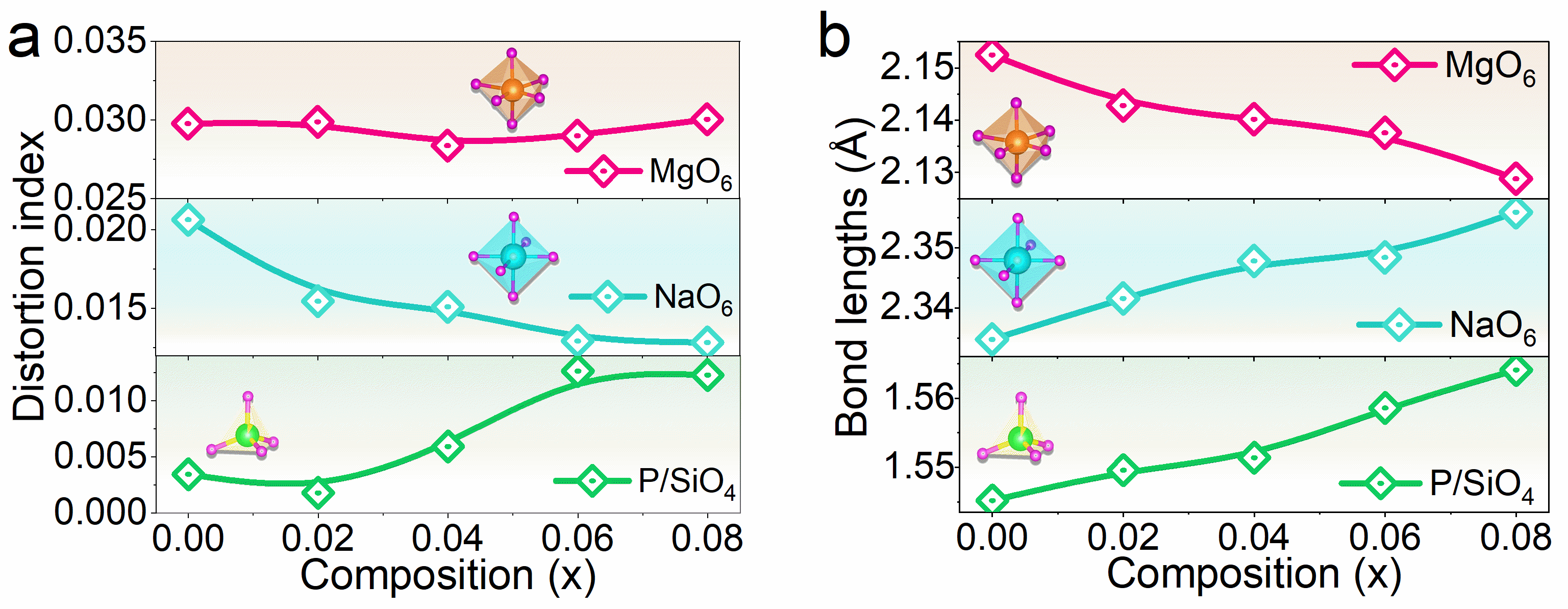


**Fig. S9** The distortion indexes **(a)** and bond lengths **(b)** of NaO_6_, MgO_6_ and PO_4_ polyhedra in Na_1+_*_x_*MgP_1-_*_x_*Si*_x_*O_4_:Eu^2+^ (*x*=0-0.08) depending on *x* values.


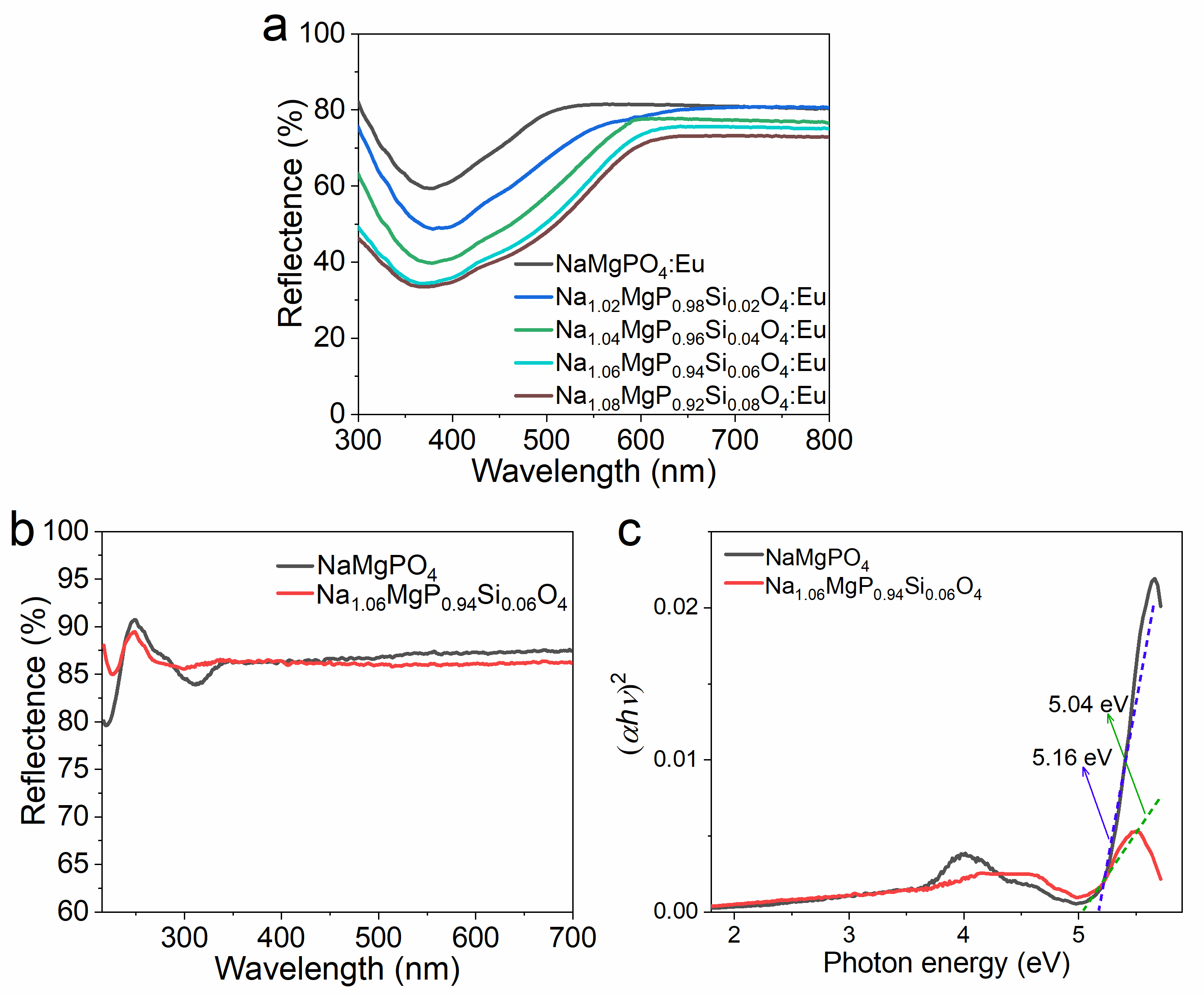


**Fig. S10** **(a)** Diffuse reflection spectra of Na_1+_*_x_*MgP_1-_*_x_*Si*_x_*O_4_:Eu^2+^ (*x*=0-0.08) phosphors (a) and NaMgPO_4_, Na_1.06_MgP_0.94_Si_0.06_O_4_ host **(b)**. **(c)** The calculated energy gap by Tauc equation for NaMgPO_4_ and Na_1.06_MgP_0.94_Si_0.06_O_4_.


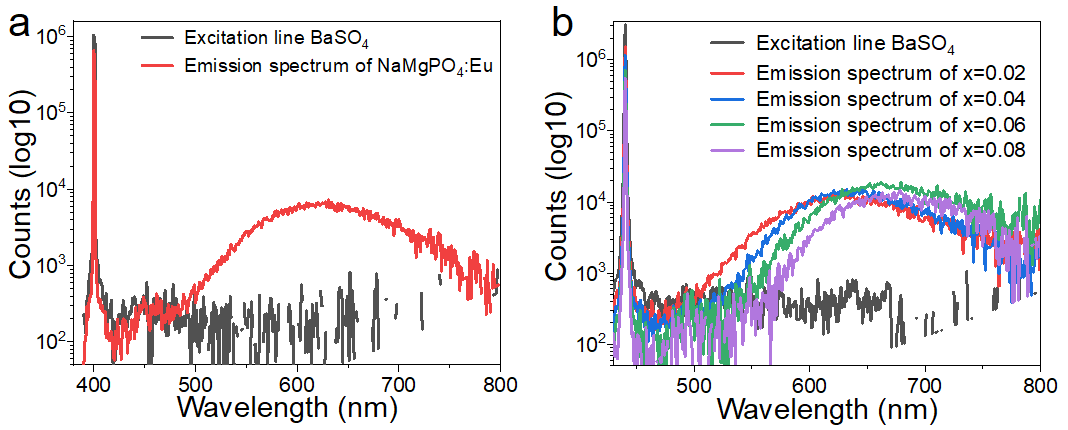


**Fig. S11** Excitation line of BaSO_4_ and the emission spectra of the NaMgPO_4_:Eu **(a)** and Na_1+_*_x_*MgP_1-_*_x_*Si*_x_*O_4_:Eu^2+^ (*x* = 0.02-0.08) phosphors **(b)** collected using an integrating sphere.

In this work, the quantum yield (QY) test was performed at room temperature by using a barium sulfate coated integrating sphere (150 mm in diameter) that attached to FLS1000. The internal quantum efficiencies (*η*) were calculated by using the following equations:

$$\eta=\frac{\varepsilon}{\alpha}=\frac{\int L_{S}}{\int E_{R}-\int E_{S}}$$

where *ε* is the number of photons emitted by the sample, and *α* is the number of photons absorbed by the sample. *L_S_* is the luminescence emission spectrum of the sample; *E_S_* is the spectrum of the light used for exciting the sample; *E_R_* is the spectrum of the excitation light without the sample in the sphere; and all the spectra were collected using the sphere.


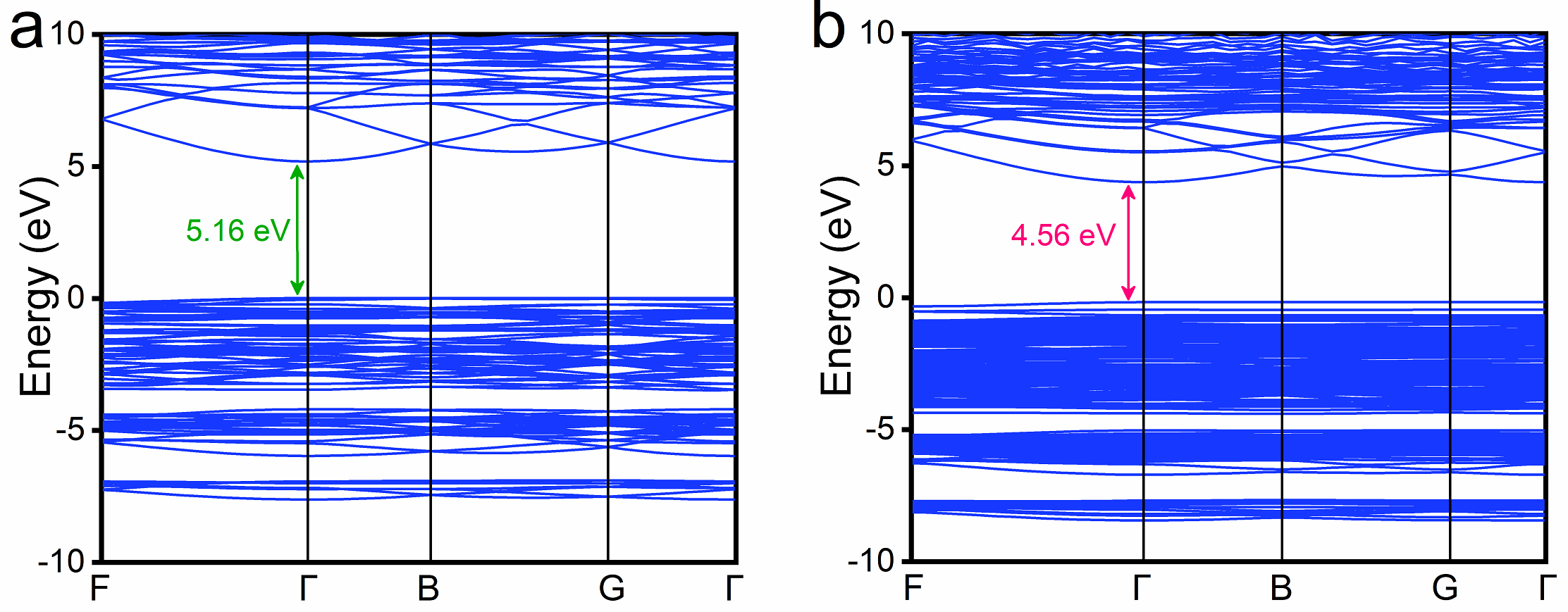


**Fig. S12** Band structure of NaMgPO_4_ **(a)** and Na_1.06_MgP_0.94_Si_0.06_O_4_ **(b)** host.


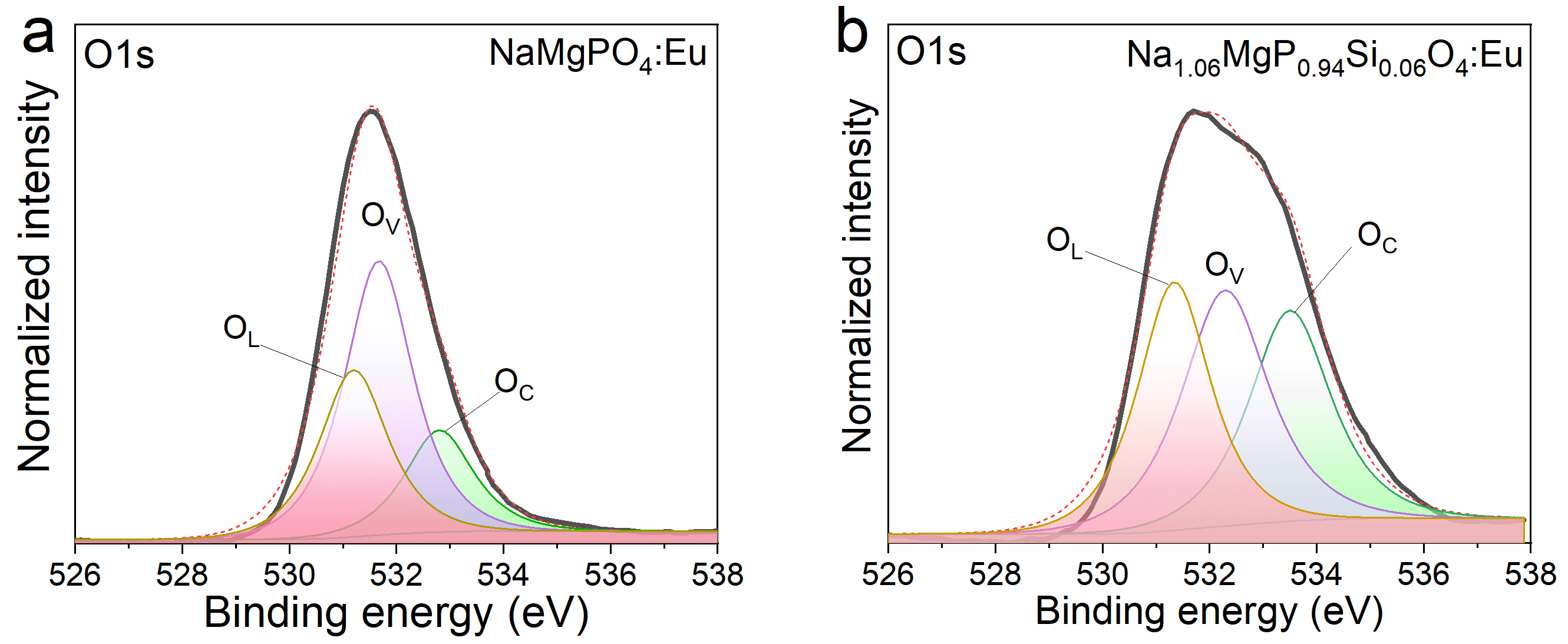


**Fig. S13** O1s XPS spectra of NaMgPO_4_:Eu^2+^(**a**) and Na_1.06_MgP_0.94_Si_0.06_O_4_:Eu^2+^ (**b**).


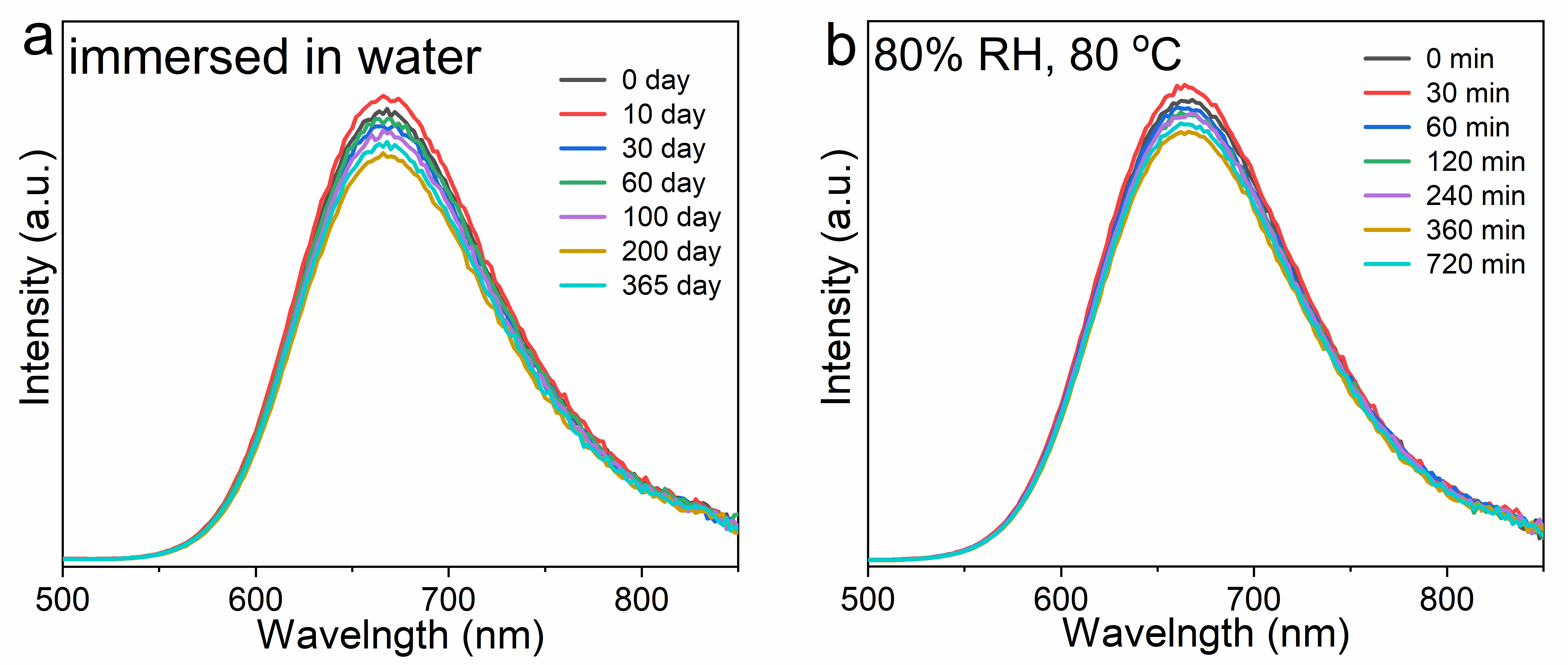


**Fig. S14** **(a)** The PL spectra of Na_1.06_MgP_0.94_Si_0.06_O_4_:Eu^2+^ when immersed in water for 0-365 days. **(b)** The PL spectra of the pristine Na_1.06_MgP_0.94_Si_0.06_O_4_:Eu^2+^ and the samples treated in degradation conditions at 80% relative humidity (RH) and 80 ^o^C for different time, respectively.

**Table S1** Main parameters of processing and refinement of the Na_1+_*_x_*MgP_1-_*_x_*Si*_x_*O_4_:Eu (*x* = 0-0.08) samples.

| *x* | 0 | 0.02 | 0.04 | 0.06 | 0.08 |
| --- | --- | --- | --- | --- | --- |
| Sp. Gr. | *Pnma* | *Pnma* | *Pnma* | *Pnma* | *Pnma* |
| *a*, Å | 10.27008(18) | 10.26847(23) | 10.27376(15) | 10.27793(17) | 10.27195(16) |
| *b*, Å | 6.18430(10) | 6.18614(12) | 6.183361(87) | 6.18196(11) | 6.186442(93) |
| *c*, Å | 4.936995(86) | 4.93706(11) | 4.938817(74) | 4.939748(91) | 4.939643(80) |
| *V*, Å^3^ | 313.5648(93) | 313.613(12) | 313.7450(80) | 313.8603(96) | 313.8986(85) |
| *Z* | 4 | 4 | 4 | 4 | 4 |
| *R_wp_*, % | 5.245 | 4.595 | 4.868 | 6.548 | 4.507 |
| *R_p_*, % | 3.824 | 3.526 | 3.71 | 4.718 | 3.483 |
| *R_exp_*, % | 2.838 | 2.843 | 2.840 | 2.873 | 2.891 |
| *χ^2^* | 1.848 | 1.616 | 1.714 | 2.279 | 1.559 |
| *R_B_*, % | 2.682 | 2.125 | 2.252 | 3.867 | 2.259 |

**Table S2** Fractional atomic coordinates and isotropic displacement parameters (Å^2^) of Na_1+_*_x_*MgP_1-_*_x_*Si*_x_*O_4_:Eu (*x* = 0-0.08) samples.

| Atom | *x* | *y* | *z* | *B*_iso_ | *Occ.* |
| --- | --- | --- | --- | --- | --- |
| *x* = 0 | | | | | |
| Na | 0 | 0 | 0 | 0.698 | 1 |
| Mg | 0.28140(11) | 0.25 | 0.99838(27) | 0.2 | 1 |
| P | 0.10956(11) | 0.25 | 0.44465(19) | 0.469(27) | 1 |
| O1 | 0.11091(23) | 0.25 | 0.75769(41) | 0.2 | 1 |
| O2 | 0.46627(23) | 0.25 | 0.15088(40) | 0.2 | 1 |
| O3 | 0.17633(18) | 0.05264(25) | 0.31232(27) | 0.3503 | 1 |
| *x* = 0.02 | | | | | |
| Na | 0 | 0 | 0 | 0.6978 | 1 |
| Na_insert_ | 0.06118(31) | 0.5630(37) | 0.0229(52) | 0.6978 | 0.01 |
| Mg | 0.28103(13) | 0.25 | 0.99998(33) | 0.2 | 1 |
| P | 0.11014(13) | 0.25 | 0.44488(24) | 0.577(35) | 0.98 |
| Si | 0.11014(13) | 0.25 | 0.44488(24) | 0.577(35) | 0.02 |
| O1 | 0.11700(29) | 0.25 | 0.75794(48) | 0.2 | 1 |
| O2 | 0.46615(28) | 0.25 | 0.15279(48) | 0.2 | 1 |
| O3 | 0.17632(23) | 0.05216(31) | 0.31105(34) | 0.3503 | 1 |
| *x* = 0.04 | | | | | |
| Na | 0 | 0 | 0 | 0.6978 | 1 |
| Na_insert_ | 0.06205(23) | 0.5652(46) | 0.0234(36) | 0.6978 | 0.02 |
| Mg | 0.28163(11) | 0.25 | 0.99880(28) | 0.2 | 1 |
| P | 0.11002(11) | 0.25 | 0.44401(20) | 0.568(27) | 0.96 |
| Si | 0.11002(11) | 0.25 | 0.44401(20) | 0.568(27) | 0.04 |
| O1 | 0.11756(24) | 0.25 | 0.75427(41) | 0.2 | 1 |
| O2 | 0.46644(23) | 0.25 | 0.15241(40) | 0.2 | 1 |
| O3 | 0.17788(19) | 0.05180(26) | 0.30858(29) | 0.3503 | 1 |
| *x* = 0.06 | | | | | |
| Na | 0 | 0 | 0 | 0.6978 | 1 |
| Na_insert_ | 0.0623(27) | 0.5668(35) | 0.0202(60) | 0.6978 | 0.03 |
| Mg | 0.28158(14) | 0.25 | 0.99768(35) | 0.2 | 1 |
| P | 0.10790(13) | 0.25 | 0.44265(26) | 0.557(33) | 0.94 |
| Si | 0.10790(13) | 0.25 | 0.44265(26) | 0.557(33) | 0.06 |
| O1 | 0.11462(31) | 0.25 | 0.74988(52) | 0.2 | 1 |
| O2 | 0.4600(30) | 0.25 | 0.14957(51) | 0.2 | 1 |
| O3 | 0.17690(24) | 0.05285(32) | 0.30557(32) | 0.3503 | 1 |
| *x* = 0.08 | | | | | |
| Na | 0 | 0 | 0 | 0.6978 | 1 |
| Na_insert_ | 0.0621(17) | 0.5672(28) | 0.0205(45) | 0.6978 | 0.04 |
| Mg | 0.28191(10) | 0.25 | 0.99811(26) | 0.2 | 1 |
| P | 0.108041(99) | 0.25 | 0.44384(18) | 0.392(25) | 0.92 |
| Si | 0.108041(99) | 0.25 | 0.44384(18) | 0.392(25) | 0.08 |
| O1 | 0.12098(22) | 0.25 | 0.75518(38) | 0.2 | 1 |
| O2 | 0.46465(22) | 0.25 | 0.15137(37) | 0.2 | 1 |
| O3 | 0.17894(18) | 0.05108(24) | 0.30621(27) | 0.3503 | 1 |

**Table S3** Main bond lengths (d, Å), volumes (V, Å^3^) and Distortion index (Dis) of NaO_6_, MgO_6_ and PO_6_ polyhedra in Na_1+_*_x_*MgP_1-_*_x_*Si*_x_*O_4_:Eu (*x* = 0-0.08) crystal structure.

|  | Na-d (Å) | Na-V (Å^3^) | Na-Dis | Mg-d (Å) | Mg-V (Å^3^) | Mg-Dis | P-d (Å) | P-V (Å^3^) | P-Dis |
| --- | --- | --- | --- | --- | --- | --- | --- | --- | --- |
| *x*=0 | 2.3348 | 14.9496 | 0.0206 | 2.1525 | 12.3133 | 0.0297 | 1.5452 | 1.8802 | 0.0034 |
| *x*=0.02 | 2.3416 | 15.0593 | 0.0154 | 2.1428 | 12.1661 | 0.0298 | 1.5496 | 1.8981 | 0.0018 |
| *x*=0.04 | 2.3478 | 15.2077 | 0.0151 | 2.1402 | 12.1246 | 0.0283 | 1.5514 | 1.9046 | 0.0059 |
| *x*=0.06 | 2.3484 | 15.3905 | 0.0129 | 2.1376 | 12.0902 | 0.029 | 1.5586 | 1.9301 | 0.0126 |
| *x*=0.08 | 2.3559 | 15.4032 | 0.0128 | 2.1288 | 11.9356 | 0.0300 | 1.5641 | 1.9513 | 0.0123 |

**Table S4** The crystal radii and ionic radii of Eu^2+^, Na^+^, Mg^2+^, P^5+^, Si^4+^ in the different fold of coordination.

| **Ion** | **Coordination number** | **Crystal radii (Å)** | **Ionic radii (****Å)** |
| --- | --- | --- | --- |
| Na^+^ | 6 | 1.16 | 1.02 |
| Mg^2+^ | 6 | 0.86 | 0.72 |
| P^5+^ | 4 | 0.31 | 0.17 |
| Si^4+^ | 4 | 0.4 | 0.26 |
| Eu^2+^ | 6 | 1.31 | 1.17 |

**Table S5** Luminescent decay times (τ_1_, τ_2_) and the fitting constants (A_1_, A_2_) of the fast and slow components, average decay times (τ_av_) and standard error of τ_av_. These decay times were measured by FLS1000 equipped with 450 nm pulse laser diode as excitation. All the datas were fitted by the Origin software.

| λ_ex_= 440 nm, Temperature = 300 K | | | | | | | |
| --- | --- | --- | --- | --- | --- | --- | --- |
| *x* | A1 | τ_1_ | A_2_ | τ_2_ | τ_av_ (μs) | R^2^ |  |
| 0 | 15570.63 | 1.943 | 13019.06 | 2.582 | 2.27 | 0.9997 |  |
| 0.02 | 15160.08 | 2.128 | 10138.45 | 2.605 | 2.34 | 0.9998 |  |
| 0.04 | 19813.94 | 2.112 | 17565.382 | 2.713 | 2.43 | 0.9996 |  |
| 0.06 | 13078.69 | 1.851 | 13886.94 | 2.906 | 2.51 | 0.9997 |  |
| 0.08 | 25110.06 | 2.414 | 1219.99 | 4.923 | 2.58 | 0.9996 |  |
| λ_ex_= 440 nm, Temperature = 78 K | | | | | | | |
| 0 | 40067.25 | 2.339 | 1227.36 | 4.426 | 2.45 | 0.9997 |  |
| 0.04 | 25412.04 | 2.373 | 1388.18 | 4.232 | 2.53 | 0.9998 |  |
| 0.08 | 40499.6 | 2.357 | 4225.87 | 4.083 | 2.62 | 0.9998 |  |

Average decay times are calculated by the following question:

$\tau_{av}=\frac{A_{1}\tau_{1}^{2}+A_{1}\tau_{2}^{2}}{A_{1}\tau_{1}+A_{2}\tau_{2}}$

Where *A_1_* and *A_2_* are the corresponding fitting constants; $\tau_{1}$, $\tau_{2}$ are lifetimes for the fast and slow components.

**Table S6** Hirshfeld surface analysis calculates the NaO_6_ and MgO_6_ as the center, volume, surface area and the contribution of P-O and Si-O to the HS surface in Na_1+_*_x_*MgP_1-_*_x_*Si*_x_*O_4_:Eu (*x* = 0-0.08).

|  | Na-V  (Å^3^) | Na-A  (Å^2^) | Na-[ P-O]  (%) | Na-[O-Si]  (%) | Mg-V  (Å^3^) | Mg-A  (Å^2^) | Mg-[P-O]  (%) | Mg-[Si-O]  (%) |
| --- | --- | --- | --- | --- | --- | --- | --- | --- |
| *x*=0 | 56.53 | 106.26 | 33.7 | 0 | 61.85 | 94.33 | 38.5 | 0 |
| *x*=0.02 | 56.82 | 106.65 | 22.1 | 12.3 | 61.70 | 93.92 | 12.0 | 26.5 |
| *x*=0.04 | 57.04 | 107.06 | 20.9 | 12.4 | 61.71 | 93.80 | 11.9 | 26.4 |
| *x*=0.06 | 57.29 | 107.47 | 20.7 | 12.5 | 61.80 | 93.77 | 11.6 | 26.2 |
| *x*=0.08 | 57.49 | 107.84 | 20.5 | 12.4 | 61.68 | 93.34 | 11.6 | 26.3 |

**References**

1. Kim, S. W. *et al.* Efficient red emission of blue-light excitable new structure type NaMgPO_4_:Eu^2+^ phosphor. *ECS Solid State Lett.* **2**, R49 (2013).

2. Hasegawa, T. *et al.* Phase stabilization of red-emitting olivine-type NaMgPO_4_:Eu^2+^phosphorsviamolten-phase quenching. *Inorg. Chem. Front.* **7**, 4040-4051 (2020).
